# Reinforcement Learning–Based Temporal Knowledge Graph Reasoning for Predicting Chronic Gastritis Diagnosis and Treatment: Development and Validation Study

**DOI:** 10.2196/83544

**Published:** 2026-09-11

**Authors:** Xiaolong Qu, Zhou Sun, Yuhang Wang, Haiyu Liu, Lei Yao, Dongmei Li, Guanli Song, Runshun Zhang, Xiaoping Zhang

**Affiliations:** 1 School of Information Science and Technology, Beijing Forestry University, 35 Qinghua East Road, Haidian District, Beijing, 100091, China, 86 13120253485; 2 Hebei Key Laboratory of Smart National Park, Beijing, China; 3 National Data Center of Tradition Chinese Medicine of China, Academy of Chinese Medical Sciences, Beijing, China; 4 University of Wisconsin–Milwaukee, Milwaukee, WI, United States

**Keywords:** temporal knowledge graph reasoning, reinforcement learning, chronic gastritis, diagnosis prediction, traditional Chinese medicine

## Abstract

**Background:**

The clinical progression of chronic gastritis involves intricate temporal dependencies, which makes it difficult to capture both the dynamic trajectory of the disease and the underlying relationships among medical events using conventional methods.

**Objective:**

This study aims to dynamically predict chronic gastritis diagnosis. We propose RL4TKGR, a reinforcement learning–based temporal knowledge graph (TKG) reasoning model, to predict a chronic gastritis diagnosis.

**Methods:**

RL4TKGR incorporates a reinforcement learning policy network that uses a dual-path encoding module to model historical and nonhistorical diagnostic information separately. RL4TKGR further integrates a dual-channel reward function with a dynamic weight allocation mechanism, which adaptively balances the two information sources. This design addresses the strategic bias problem and enables interpretable reasoning. The chronic gastritis temporal knowledge graph (CG-TKG) was constructed from chronic gastritis diagnosis and treatment records of 17,906 patients comprising 38,360 visits from March 2009 to October 2022.

**Results:**

Experiments on the self-constructed CG-TKG resulted in RL4TKGR achieving the highest mean reciprocal rank (MRR) on CG-TKG-4 (51.66, SD 0.76), CG-TKG-8 (53.18, SD 0.79), and CG-TKG-12 (56.95, SD 0.84). After adding recent TKG reasoning baselines, adaptive path-memory network (DaeMon) achieved the strongest baseline MRR for all 3 subsets, with MRR values of 43.26 (SD 0.95), 51.76 (SD 1.12), and 54.87 (SD 1.17), respectively. Compared with DaeMon, the overall paired *t* tests across MRR and Hits@1/3/10 yielded 𝑃<.001 for all 3 subsets. Ablation experiments and case analyses further supported the contribution of the historical modeling components and the practical utility of the model for predicting disease subtypes and therapeutic medications.

**Conclusions:**

This study improves chronic gastritis diagnosis and treatment prediction by integrating a history-aware dual-path policy network with a dynamic event balance factor in reinforcement learning–based TKG reasoning.

## Introduction

### Background

Chronic gastritis is a chronic inflammation of the gastric mucosa caused by multiple pathogenic factors. Its spectrum of precancerous lesions, including gastric atrophy, intestinal metaplasia, and dysplasia, has conferred independent carcinogenic risks [1]. Consequently, elucidating the evolutionary trajectory of chronic gastritis and accurately predicting its progression are of critical importance for the early detection and intervention of gastric cancer, which underscores the significant clinical value of this task.

In recent years, artificial intelligence (AI) technologies have been extensively explored for the diagnosis and treatment of chronic gastritis. In prediction studies based on static features, several models were used to analyze gastroscopic images and demonstrated high accuracy and efficiency in the diagnosis of gastric diseases such as chronic atrophic gastritis, which significantly improved the accuracy of real-time diagnosis [2-4]. In the study by Tao et al [5], the model not only was able to diagnose gastric mucosal atrophy but also performed risk stratification based on the extent of atrophy; this provided decision support for subsequent monitoring intervals. However, static prediction models struggle to capture the dynamic evolution trends of diseases and fail to adequately consider the temporal characteristics inherent in clinical progression. Therefore, current research has gradually shifted from static, point-in-time diagnosis toward dynamic risk prediction and disease course evolution analysis based on temporal medical data. Existing studies leveraged electronic medical record (EMR) data and applied temporal analysis techniques to predict the progression risk from atrophic gastritis to gastric cancer while also quantifying relevant disease trajectories and high-risk factors [6,7]. Nevertheless, temporal medical data primarily record sequences of indicator values changing over time and inherently lack the capacity to organize information into structured tuples. This limitation constrains the ability to model deeper associations among medical events.

Temporal knowledge graphs (TKGs) extend the static knowledge graph framework by introducing the temporal dimension [8], and they represent the evolutionary processes of dynamic events using quadruples of the form (s,p,o,t). In medical scenarios, knowledge graphs can provide physicians with reliable auxiliary diagnostic tools and offer good explainability [9]. Moreover, TKGs can model the dynamic evolution of relationships between entities and demonstrate key value for disease progression prediction and drug interaction tracking [10]. Static pathological features are limited in capturing the dynamic trends of disease progression and overlook the clinical evolution of the disease course, whereas TKGs can embed time stamps into entity states to capture the progressive nature of chronic gastritis. In contrast to temporal data, which capture variations in indicator values over time but cannot organize information into structured tuples, TKGs enable the representation of the clinical evolution of chronic gastritis, thereby improving both the accuracy and interpretability of prediction. Nevertheless, studies that leverage TKGs to predict the dynamic progression of chronic gastritis remain scarce.

In clinical practice, historical diagnostic information for patients plays an essential role in predicting diagnoses for subsequent patients with the same disease. The patterns of disease representation similarity in patient symptoms [11] provide a theoretical foundation for temporal diagnosis and treatment modeling. Reinforcement learning excels at deriving optimal strategies through interaction with the environment, which makes it well suited to simulate the clinical decision-making process in which physicians formulate future decisions based on the historical medical records and current conditions of patients. Furthermore, the medical decision-making process can be regarded as a sequential decision-making problem of finding the optimal path within massive information, which aligns closely with the modeling paradigm of reinforcement learning. Therefore, we constructed a chronic gastritis temporal knowledge graph (CG-TKG) based on real-world dynamic diagnostic and treatment data. By modeling and analyzing the historical medical records of patients and incorporating the characteristics of clinical data, we proposed a reinforcement learning–based TKG reasoning model named RL4TKGR. The main methodological novelty of RL4TKGR lies in its architecture-specific separation of historical and nonhistorical diagnostic paths and its dynamic β-based weighting mechanism, rather than in the general use of TKG reasoning or reinforcement learning alone. This model is designed to improve prediction of disease subtypes and therapeutic drugs for chronic gastritis on the evaluated CG-TKG task.

To summarize, the contributions of this paper are as follows: (1) We proposed RL4TKGR for chronic gastritis diagnosis prediction. We designed a reinforcement learning policy network that exploits associations within historical diagnostic data to predict chronic gastritis diagnosis. RL4TKGR captures both the features from the historical medical records of patients and the latent features of nonhistorical diagnoses, which enables interpretable reasoning on the CG-TKG. (2) We proposed a dual-channel aggregated reward function and a dual-path encoded policy network based on historical diagnosis. By constructing reward functions for historical and nonhistorical information, the model guided the learning of the corresponding paths within the policy network. Furthermore, the event balance factor was used to dynamically and interpretably adjust the weight allocation in the dual-channel aggregation reward function and the action scoring module, which effectively addressed the strategy bias issue caused by static weight allocation. (3) On the self-constructed CG-TKG datasets, RL4TKGR achieved mean reciprocal rank (MRR) values of 51.66 on CG-TKG-4, 53.18 on CG-TKG-8, and 56.95 on CG-TKG-12, compared with the strongest MRR baselines of 43.26, 51.76, and 54.87, respectively, after adding recent TKG reasoning baselines. RL4TKGR also achieved Hits@10 values of 60.15, 67.26, and 71.79 on the 3 datasets, respectively. These absolute performance values indicated that RL4TKGR improves chronic gastritis diagnosis and treatment prediction on temporal clinical data.

### Related Work

#### TKG Reasoning

A TKG is a special type of knowledge graph that adds a temporal dimension to the traditional triple representation (entity, relation, entity), which forms a new quadruple (entity, relation, entity, time). This representation can capture the temporal evolution of relationships among entities, which is of great significance for understanding and predicting their dynamic interactions [12].

The TTransE [13] model extends the TransE [14] model by adding a temporal dimension. Its scoring function considers temporal information, which enables the model to handle quadruples in TKGs. The TA-DistMult [15] model is a temporal version of the DistMult [16] model that integrates temporal information by learning a sequence encoder. This model uses a recurrent neural network (RNN) to learn time-aware representations of relation types. Similarly, TA-TransE [15] is a temporal variant of TransE that incorporates temporal information into the standard embedding framework for link prediction. This model leverages an RNN to encode sequences of temporal tokens, thereby learning time-aware representations.

DE-SimplE [17] equips static models with diachronic entity embedding for TKG completion. xERTE [18] proposes an explainable reasoning framework for future link prediction in TKGs, which uses a temporal relation attention mechanism and a novel reverse representation update scheme. TimeTraveler (TITer) [19] was the first to use path-based reinforcement learning for TKG reasoning, and it can handle unseen time stamps and entities. In addition, run-time type information (RTTI) [20] proposed a new method for representing time intervals on this basis, which uses the median of 2 time stamps and embedding changes to represent time intervals.

Existing works adopt various TKG extrapolation architectures to model the evolution patterns of TKGs. Relation-enhanced graph convolutional network (RE-GCN) [21] designs a recurrent evolutionary network to capture both structural dependencies and temporal patterns. To address the problem of length diversity in evolution patterns, complex evolutional network (CEN) [22] combines relational graph neural networks and length-aware convolutional networks to learn evolution features of historical sequences of different lengths. Recurrent event network (RE-Net) [23] models the temporal conditional probability distribution of event sequences through a recursive event encoder, while it uses a neighborhood aggregator to handle concurrent events and enable global structural reasoning. Contrastive event network (CENET) [24] further considers the influence of potential unobserved factors and leverages contrastive learning to model dependencies between historical and nonhistorical events, thereby improving predictive performance.

To avoid grouping heterogeneous methods into a single category, recent TKG reasoning studies can be organized into several families. Time-aware embedding models, such as TTransE, TA-DistMult, TA-TransE, and DE-SimplE, encode temporal signals into entity or relation representations. Continuous-time models, such as Know-Evolve [25] and Temporal Knowledge Graph Forecasting with Neural Ordinary Equations (TANGO) [26], model event intensities or continuous dynamic embeddings rather than only discrete graph snapshots. Recurrent and graph-based extrapolation architectures, including RE-Net, RE-GCN, CEN, CENET, time-guided recurrent graph network (TiRGN) [27], relation-entity twin-interact aggregation (RETIA) [28], and adaptive path-memory network (DaeMon) [29], emphasize future-link prediction from historical snapshots. Path-based and rule-based models, such as TITer, xERTE, and TLogic [30], provide more explicit reasoning paths or temporal logical rules. RL4TKGR belongs to the history-aware path reasoning family and is tailored to discrete visit-order clinical TKGs.

Although these model families have advanced TKG reasoning, several limitations remain for visit-level clinical TKGs. Embedding and recurrent extrapolation models improve temporal prediction but often treat historical facts as global graph snapshots and provide limited patient-level path evidence. Continuous-time models can represent irregular event times, but they require reliable time-stamped event streams and are less directly aligned with deidentified visit-order clinical records. Path-based methods improve interpretability, yet existing designs generally do not explicitly separate historical diagnostic paths from current or nonhistorical clinical evidence. These gaps motivate the dual-path policy network and dynamic event balance factor in RL4TKGR.

#### Application of TKG Reasoning in Disease Prediction

Knowledge graph reasoning technology in the medical field is evolving from static modeling to dynamic temporal analysis [31]. Carvalho et al [32] proposed an ontology-based framework for constructing EMR temporal graphs that achieved dynamic tracking of patient status through semantic annotation and time stamp mapping and established a foundation for the advancement of knowledge graph reasoning in the medical domain.

Considering the temporal characteristics of diabetes progression, Geng et al [33] proposed an incremental long short-term memory (LSTM) that embeds entity relations through TransR, integrates graph topological structures, and achieved precise modeling of complication evolution on clinical knowledge graphs. Chaturvedi [34] leveraged a pretrained transformer to extract fine-grained temporal relations from clinical texts and construct a patient-centered multisource temporal graph that provides a scalable risk prediction framework for chronically monitored diseases such as type 2 diabetes. Considering the task characteristics of long-term health risk prediction, Postiglione et al [35] proposed the MedTKG framework, which integrates the dynamic temporal information from electronic health records (EHRs) with the static hierarchy of medical ontologies and validates the effectiveness of ontology-enhanced temporal reasoning on the MIMIC-III dataset. All of these efforts focused on chronic disease evolution and complication prediction. For the prediction of acute disease deterioration, Song et al [10] accounted for the rapid worsening of disease trajectories by integrating temporal information into a gated recurrent unit (GRU) network while preserving graph structural properties with TransR, thereby developing a diagnostic support system capable of real-time prediction of acute diseases and their complications.

However, most medical TKG applications focus on general EHR risk prediction, complications, or acute deterioration, and few address chronic gastritis diagnosis and treatment prediction in a traditional Chinese medicine (TCM)–specific entity space. In addition, existing clinical graph studies often emphasize predictive accuracy or ontology construction, whereas fewer studies examine how historical and current-visit evidence are balanced for individual patients.

#### Application of Reinforcement Learning in Disease Prediction

As one of the 3 major paradigms of machine learning, reinforcement learning centers on the dynamic interaction between an agent and its environment, optimizing decision-making by maximizing target objectives through reward signals. Unlike supervised learning, reinforcement learning models the decision-making trajectory through a Markov decision process [36].

The core advantage of reinforcement learning in disease prediction lies in its dynamic decision optimization capability. Compared with static prediction models, reinforcement learning can simulate the multistage process of disease development and formulate personalized intervention strategies according to the real-time status of patients. In the context of Alzheimer disease prediction, Chaudhari and Khot [37] proposed CAdam-RL-DCNN, which integrates an improved Coyote optimization algorithm with the Adam optimizer and combines a reinforcement learning decision mechanism to address the problems of feature extraction efficiency and class imbalance. Zhang et al [38] constructed a weighted dueling double deep Q-network, integrated clinical expert rules to guide action selection, and ensured decision reliability through doubly robust off-policy evaluation, providing a new paradigm for dynamic treatment in intensive care units.

Despite these advances, most existing TKG reasoning models in the medical domain rely on static weight fusion or fixed embedding schemes, which limits their ability to dynamically adapt to individualized diagnostic and treatment trajectories or to differentiate historical and nonhistorical clinical events, thus leading to policy bias. Likewise, current reinforcement learning–based disease prediction models often depend on static rules or weight fusion, which constrains their capacity to dynamically capture the relevance of historical events. To address these challenges, we proposed the RL4TKGR model based on historical diagnostic information. This model effectively overcomes the bias problem of static fusion and significantly improves the accuracy and interpretability of chronic gastritis prediction.

## Methods

### Model Framework

**Figure 1.**
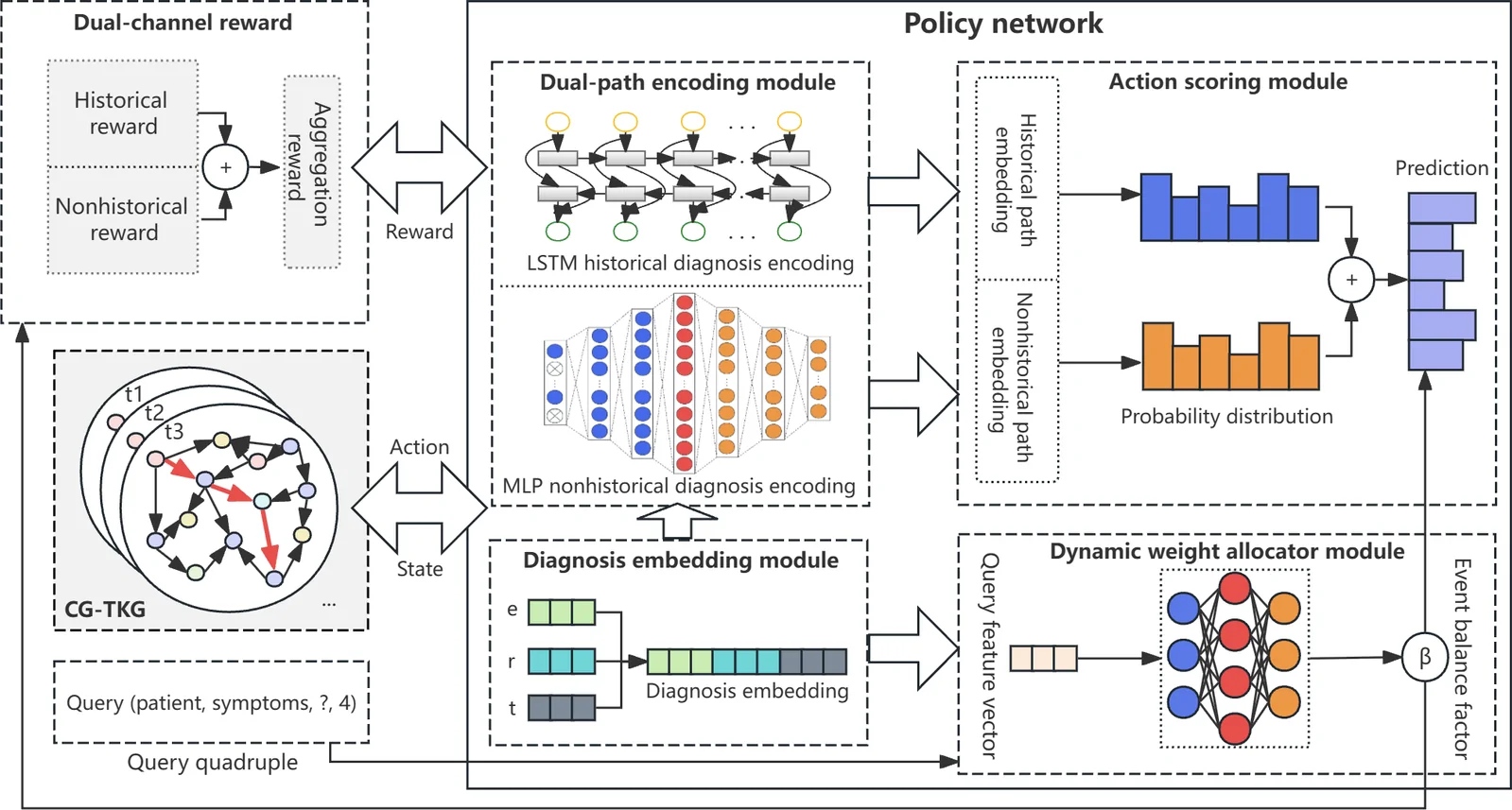
Framework of the proposed reinforcement learning–based temporal knowledge graph reasoning (RL4TKGR) model. CG-TKG: chronic gastritis temporal knowledge graph; LSTM: long short-term memory.

The framework of RL4TKGR is shown in Figure 1, with the policy network as its core component. The policy network consists of 4 modules: diagnosis embedding module, dual-path encoding module, action scoring module, and dynamic weight allocator module. The dual-channel reward function guides the learning of the policy network. It comprises both a historical reward function and a nonhistorical reward function, which are adaptively integrated through the dynamic weight allocation module.

### Ethical Considerations

This study was approved by the Medical Ethics Committee of Guang’anmen Hospital, China Academy of Chinese Medical Sciences (approval number: 2024‐241-KY; approval date: December 10, 2024; valid from December 10, 2024, to December 31, 2027). The ethics committee approved the study protocol and granted a waiver for informed consent. Only deidentified clinical records were used for model development and validation, and the research team has no access to direct patient identifiers. Individual-level clinical records are not publicly shared because they contain sensitive health information and are subject to institutional ethics and data governance restrictions.

### Formal Definitions

#### TKG

A TKG was defined as $G_{\left(1,T\right)}=\left(G_{1},G_{2},\ldots ,G_{T}\right)$, where $G_{t}=\left(E_{t},R_{t},F_{t}\right)$ denotes a snapshot at time stamp t. Here, $E_{t}$ represents the entity set, $R_{t}$ represents the relation set, and $F_{t}$ represents the fact set at t. Each fact is expressed as $F_{t}=\left(e_{s},r,e_{o},t\right)$, where $e_{s},e_{o}\in E_{t}$ indicate subject and object entities, respectively, and $r\in R_{t}$ denotes their relation. In the CG-TKG, t denotes the visit number, $E_{t}$ denotes the set of medical entities at the t-th visit, $R_{t}$ denotes the set of medical relations at the t-th visit, and $F_{t}$ denotes the set of medical facts at the *t*-th visit.

In the CG-TKG, time stamp *t* is encoded as the visit-order index rather than continuous calendar time. Thus, *t*=4 denotes the fourth recorded clinical visit of a patient, not a specific date. This discrete encoding matches the visit-level organization of the clinical records and supports temporal snapshot construction. However, it does not directly model heterogeneous elapsed days between adjacent visits.

#### TKG Reasoning Task

The TKG reasoning task was defined as performing link prediction in knowledge graphs at future time stamps along the temporal evolution. Given a query), the missing object entity in the query is predicted using the TKG composed of the set of known facts . In the CG-TKG reasoning task, the query) indicates that the missing medical entity is predicted based on the set of known medical facts in the CG-TKG, with a primary focus on predicting the types of chronic gastritis and corresponding therapeutic drugs. For example, the query (patient ID,diagnosis,…,4) indicates predicting the disease diagnosis of a patient (identified by patient ID) at the fourth clinical visit based on the set of medical facts from the first 3 visits.

### Reinforcement Learning Framework

#### States

We defined S as the state space, where each state can be represented as a 5-tuple $s_{n}=\left(e_{n},t_{n},e_{q},t_{q},r_{q}\right)\in S$. Here, $\left(e_{n},t_{n}\right)$ denotes the node accessed at step n and visible exclusively at step n, with $t_{n}§amp;lt;t_{q}$; $\left(e_{q},t_{q},r_{q}\right)$ representing elements in the query $\left(e_{q},r_{q},\ldots ,t_{q}\right)$ and globally visible. The agent uses $s_{0}=\left(e_{q},t_{q},e_{q},t_{q},r_{q}\right)$ as the initial state.

#### Actions

We defined A as the action space, and $A_{n}$ as the set of optional actions at step n, where $A_{n}=\left\{\left(r^{\prime },e^{\prime },t^{\prime }\right)\vee \left(e_{n},r^{\prime },e^{\prime },t^{\prime }\right)\in F_{t^{\prime }},t^{\prime }<t_{q},t_{n}<t_{q}\right\}$. Since a patient’s disease progresses with each visit, the agent can select actions with time stamps subsequent to $t_{n}$ during action selection. Therefore, in the TKG reasoning task, the agent can select actions from the entire set of known facts.

#### Transition

We defined a state transition function δ to map from state $S_{n}$ to $S_{n+1}$ during the state transition performed by the agent, such that S×A→S (ie, $S_{n+1}=\delta \left(s_{n},a_{n}\right)=\left(e_{n+1},t_{n+1},e_{q},t_{q},r_{q}\right)$.

#### Rewards

We defined a dual-channel aggregated reward function R that integrates historical and nonhistorical diagnostic information to guide the agent toward accurate action selection.

### Dual-Channel Reward Function

#### Historical Reward

The historical reward enables the policy network to focus on historical diagnosis information. When the predicted fact is related to historical interactions, the model incorporates these known facts into the prediction of future facts, as expressed by Equation 1:


(1) $$R_{his}(s_{L})=I\left\{e_{L}=e_{fact}\wedge e_{L}\in H_{q}\right\}\cdot \left(1+p_{his}(e_{L})\right)$$

where $H_{q}$ denotes the set of historical entities, that is, all entities that appeared in the previous n visits. The historical occurrence frequency $p_{his}$ of entity $e_{L}\in H_{q}$ is calculated and participates in the reward function computation. Here, $s_{L}$ denotes the terminal state after L reasoning steps, $e_{L}$ denotes the terminal entity reached by the selected action trajectory, $e_{fact}$ denotes the ground-truth object entity of the query, and n denotes the number of visits before the query time stamp $t_{q}$ that are available as patient history.

#### Nonhistorical Reward

The nonhistorical reward does not consider the influence of historical information in the diagnosis process and allows the predicted fact to be treated as a new potential fact unrelated to historical interactions, as expressed by Equation 2:


(2) $$R_{nhis}(s_{L})=I\left\{e_{L}=e_{fact}\right\}$$

where $e_{L}$ is the predicted entity of the action finally selected by the agent, and $e_{fact}$ is the correct entity in the query quadruple.

#### Dual-Channel Aggregation Reward

The dual-channel aggregated reward function performs a weighted integration of the historical and nonhistorical reward functions, which can be expressed as Equation 3:


(3) $$R=\alpha \cdot R_{his}+(1-\alpha )\cdot R_{nhis}$$

where α is the aggregation weight. In RL4TKGR, α is set to the query-level value of the event balance factor β generated by the dynamic weight allocator module. In the fixed- sensitivity experiment, this adaptive value was replaced by prespecified constants to isolate the effect of the historical information weight.

#### Policy Network

The policy network $\pi_{\theta }\left(a_{n}|s_{n}\right)$ evaluates the action $a_{n}\in A_{n}$ selected by the agent, where θ denotes the model parameters. This network consists of 3 modules: diagnosis embedding module, dual-path encoding module, action scoring module, and dynamic weight allocator module.

##### Diagnosis Embedding Module

We began by randomly and uniformly initializing each fact $F_{t}=\left(e_{s},r,e_{o},t\right)$ within a low-dimensional dense vector space. Since a patient’s disease progression is closely related to visit number, the entity and visit number were jointly represented as a diagnosis at the current step, that is, $e_{n}^{t}=\left(e_{n},t\right)$. Entities, relations, and time stamps are represented as $e\in R^{d_{e}}$, $r\in R^{d_{r}}$, and $t\in R^{d_{t}}$, where $d_{e}$, $d_{r}$, and $d_{t}$, respectively, denote their embedding dimensions.

During the patient’s visit process, diseases evolve gradually over time. For a prediction query $\left(e_{q},r_{q},\ldots ,t_{q}\right)$, when the agent selects an action, $\Delta t=t_{q}-t_{n}$ represents the distance between the query visit number $t_{q}$ and the current visit number $t_{n}$, which is then encoded using an activation function. Thus, the representation of a diagnosis at the current step is given by Equation 4:


(4) $$e_{n}^{t}=\left[e_{n};\sigma \left(w\Delta t+b\right)\right]$$

where w and b are learnable parameters, σ is an activation function, and [;] denotes the vector concatenation operation.

##### Dual-Path Encoding Module

The dual-path encoding module consists of the LSTM historical diagnosis encoding and the multilayer perceptron (MLP) nonhistorical diagnosis encoding.

###### LSTM Historical Diagnosis Encoding

For the historical diagnosis information for the patient, an LSTM layer encodes such information as the historical path embedding. The historical diagnosis of the patient is represented by Equation 5:


(5) $$h_{n}=\left(a_{0},a_{1},\ldots ,a_{n-1}\right)$$

where $a_{n}\in A_{n}$ denotes the action selected by the agent at step n, that is $\left(r_{n},e_{n},t_{n}\right)$. Thus, $h_{n}$ denotes the selected action sequence before step n, and $a_{0}$ denotes the first action taken from the initial state.

The term $h_{n}$ is embedded into a continuous vector space $R^{3d}$ then encoded. The historical path embedding is given by Equation 6:


(6) $$h_{n}^{his}=LSTM\left(h_{n-1},Ea_{n}\right)$$

where $E\left(a_{n}\right)$ denotes the embedding of $a_{n}$, and $h_{0}^{his}=\text{LSTM}\left(0,Ea_{0}\right)$. The superscript his denotes the hidden representation produced by the historical LSTM branch.

###### MLP Nonhistorical Diagnosis Encoding

For the nonhistorical diagnosis information for the patient, an MLP extracts latent semantic features of the nonhistorical diagnosis entities as nonhistorical path embedding. The representation of the nonhistorical diagnosis information for the patient consists of a fully connected layer and an activation function, as given by Equation 7:


(7) $$h_{n}^{nhis}=MLP\left(s;p;Ea_{n}\right)=W2\cdot ReLU\left(W1\cdot \left(s;p;Ea_{n}\right)+b1\right)+b2$$

where W1, W2, b1, and b2 are learnable parameters, $E\left(a_{n}\right)$ denotes the embedding of $a_{n}$, and [;] denotes the vector concatenation operation. s and p denote the subject entity embedding and relation embedding of the current query, respectively.

### Action Scoring Module

The policy network generates scores for each optional action and calculates the state transition probabilities. This module computes state transition probabilities jointly based on the outputs of the dual-path encoding module.

To balance the influence of historical and nonhistorical diagnoses, the event balance factor β was introduced to perform weighted allocation on their action scores. Ultimately, the probability distribution P over candidate actions is derived according to Equation 8:


(8) $$P=\beta \cdot P_{\text{his}}+\left(1-\beta \right)\cdot P_{\text{nhis}}$$

where $P_{\text{his}}$ is the state transition probability distribution of historical diagnoses and $P_{\text{nhis}}$ is the state transition probability distribution of nonhistorical diagnoses.

### Dynamic Weight Allocator Module

In the dynamic weight allocator module, the event balance factor β dynamically and interpretably adjusts the weight allocation in both the dual-channel aggregated reward function and the action scoring module.

The query q(s,p,…,t) is encoded as $v_{q}=\left[s_{q};p_{q};t_{q}\right]$, where $s_{q}$ is the embedding vector of the query entity, $p_{q}$ is the embedding vector of the query relation, and $t_{q}$ is the embedding vector of the visit number. The term $v_{q}$ is input into a fully connected layer, and the weight β is generated through activation by the sigmoid function, as given by Equation 9:


(9) $$\beta =sigmoid\left(W\cdot V_{q}+b\right)$$

where β denotes a query-level scalar output rather than an independent trainable parameter. The learnable parameters of the dynamic weight allocator are denoted by ϕ={W,b}, and these parameters are optimized together with the policy network parameters.

When the query q focuses on historical diagnoses, β tends toward 1; conversely, when the query q focuses on nonhistorical diagnoses, β tends toward 0. The event balancing factor β enables the model to adaptively and interpretably adjust the decision-making basis according to the specific conditions of the current patient.

### Training Procedure

The dynamic weight allocator parameters ϕ that generate β are optimized jointly with the policy network parameters θ. The dual-channel aggregated reward $R$ is maximized via the REINFORCE algorithm [39], such that the jointly trained policy network can be expressed as $\pi_{\theta ,\phi }\left(a_{n}|s_{n}\right)$.

With the search space length fixed as L, the joint training objective of ϕ and the policy network are optimized by maximizing the expected reward over the training sample set $F_{train}$ as given by Equation 10:


(10) $$J\left(\theta ,\phi \right)=\sum_{\left(s,p,o,t\right)\in F_{\text{train}}}\left[\sum_{a_{1},\ldots ,a_{L}∼\pi }R\right]$$

where $F_{train}$ denotes the training quadruple set, L denotes the maximum reasoning path length, and $a_{1},\ldots ,a_{L}$ denotes the sampled action trajectory drawn from the jointly trained policy $\pi_{\theta ,\phi }$.

Optimization is performed with the REINFORCE algorithm by iterating over all quadruples in $F_{train}$ and updating the parameters through the following stochastic gradient, as given by Equation 11:


(11) $$\nabla_{\theta ,\phi }J\left(\theta ,\phi \right)=E_{\pi_{\theta ,\phi }}\left[\nabla_{\theta ,\phi }log\pi_{\theta ,\phi }\left(a|s\right)\cdot R\right]$$

where *a* denotes the action sampled at the current reasoning step and s denotes the corresponding state.

In the action scoring module, the query-level β participates in the policy distribution and therefore affects the policy gradient update through ϕ. In the reward aggregation module, the generated β is substituted for α as a query-level weighting coefficient and is treated as a fixed numeric value when computing R; no separate gradient is taken through the reward calculation itself. The RL4TKGR training and inference pseudocode is provided in the RL4TKGR Training and Inference Pseudocode section in Multimedia Appendix 1.

### Dataset Description

The CG-TKG dataset used in this study was derived from the clinical diagnosis and treatment data for patients with chronic gastritis treated by a chief physician at Guang’anmen Hospital, China Academy of Chinese Medical Sciences, from March 2009 to October 2022. The dataset includes 17,906 patients with a total of 38,360 visit records (the visit number per patient ranges from 1 to 35). The source data were retrospective real-world clinical diagnosis and treatment records and included structured and semistructured information on symptoms, TCM diagnoses, Western medicine diagnoses, TCM syndrome diagnoses, laboratory tests, and gastroscopy and pathological examinations, which were used to construct temporal medical entities, relations, and visit-level time stamps in the CG-TKG. Before the dataset was provided to the research team, the source hospital and data center completed deidentification by removing direct patient identifiers and replacing original hospital identifiers with study-specific anonymous codes. The research team received and analyzed only the deidentified dataset. Because all records were obtained from a single institution and one chief physician’s clinical practice, the constructed CG-TKG may reflect institution-specific and practitioner-specific diagnostic and prescribing patterns. The data cover the aspects described in the following paragraphs.

For symptoms and TCM diagnoses, 40,993 types of information were included, including chief complaints; present illness history; and TCM inspection, auscultation-olfaction, inquiry, and palpation (eg, gastric distension, stomach pain, poor appetite, heartburn, acid regurgitation, white tongue coating, slippery pulse).

Regarding Western medicine diagnoses, the information included 119 types of chronic gastritis diseases, including chronic atrophic gastritis, chronic superficial gastritis, erosive gastritis, and *Helicobacter pylori*–associated gastritis.

TCM syndrome diagnoses included 263 types of results, such as spleen deficiency with dampness-heat syndrome, liver-stomach disharmony syndrome, spleen-stomach disharmony syndrome, liver depression and spleen deficiency syndrome, and spleen-stomach weakness syndrome.

There were a total of 59 laboratory test items such as comprehensive biochemistry, routine urine, complete blood cell analysis, routine stool, and occult blood involving 139 indicators such as white blood cells, red blood cells, urine protein, carbohydrate antigen 724, and alpha-fetoprotein and their results.

Gastroscopy and pathological examinations included 35 items (eg, active inflammation, intraepithelial neoplasia, atrophy, gastric mucosa texture, gastric mucosa color) and 56 descriptive indicators (eg, mild, moderate, normal, smooth and soft, rough mucosa).

Additional details on CG-TKG construction and quality control are provided in Multimedia Appendix 1. Briefly, entities and relations were extracted from structured and semistructured clinical fields, and each patient visit was treated as a visit-level temporal snapshot. Multisource observations recorded within the same visit share the same visit-order time stamp. Missing clinical items were not imputed nor treated as negative facts; only observed clinical facts were retained in the graph. The entity and relation schema was fully reviewed by TCM experts, and generated graph facts were further checked through sampled expert verification.

The CG-TKG dataset was partitioned by visit number. We selected patient data spanning 4, 8, and 12 visits to construct 3 temporal subsets. For each subset, the first 3, 7, and 11 visits (from cases with 4, 8, and 12 total visits, respectively) served as the training set, while the remaining visits were assigned to the validation and test sets. After manual verification by TCM experts, 3 dynamic chronic gastritis clinical datasets were obtained: CG-TKG-4, CG-TKG-8, and CG-TKG-12. Table 1 presents the statistics of the CG-TKG dataset.

**Table 1.** Chronic gastritis temporal knowledge graph (CG-TKG) dataset statistics.

Dataset	Entity count, n	Relation count, n	Training set size, n	Validation set size, n	Test set size, n	Time stamps	Time stamp interval, mean (variance)		Patient count, n
CG-TKG-4	2304	132	161,697	27,206	27,206	4	37.00 (84.94)		772
CG-TKG-8	1225	116	68,357	4797	4797	8	38.06 (88.90)		140
CG-TKG-12	868	39	36,569	3031	3031	12	30.45 (62.25)		54

The 3 temporal subsets were defined by visit course length and therefore have imbalanced patient counts: 772 patients in CG-TKG-4, 140 patients in CG-TKG-8, and 54 patients in CG-TKG-12. In particular, CG-TKG-12 represented a small long-course subgroup; therefore, results from this subset should be interpreted cautiously and require validation in larger long-term follow-up cohorts.

### Experimental Settings and Evaluation Metrics

RL4TKGR was implemented in PyTorch and initialized with uniform embeddings. The main hyperparameters were set as follows: The entity embedding dimension was 100, the relation embedding dimension was 100, the visit number embedding dimension was 20, the Adam optimizer was used for parameter optimization, the learning rate was 0.001, and the batch size was 512. All comparative and ablation experiments used the same dataset partitions and evaluation protocol described in the following paragraphs. To improve reproducibility without substantially expanding the main text, the source code and implementation scripts are publicly available through the project GitHub page [40]. The public repository provides the executable training and evaluation scripts and the implementation-level settings not expanded in the main text, including optimizer arguments, regularization options, dropout configuration, LSTM configuration, checkpointing, random-seed handling, and evaluation scripts.

The MRR and Hits@1/3/10 were used to evaluate the performance of RL4TKGR in TKG prediction tasks. The formulas and explanations of these evaluation metrics are explained in the following sections.

#### MRR

The MRR is defined as the average of the reciprocals of the ranks of the first relevant result for all queries, as shown in Equation 12:


(12) $$\text{MRR}=\frac{1}{\left|Q\right|}\sum_{i=1}^{\left|Q\right|}\frac{1}{{\text{rank}}_{i}}$$

where Q is the set of queries and ${\text{rank}}_{i}$ is the rank of the first correct answer in the i-th query.

#### Hits@k

Hits@k is defined as the hit rate of whether the correct answer appears in the top k results, as shown in Equation 13:


(13) $$\text{Hits@k}=\frac{1}{\left|Q\right|}\sum_{i=1}^{\left|Q\right|}1\left({\text{rank}}_{i}\leq k\right)$$

where 1(·) is an indicator function that takes 1 if the condition in the parentheses is satisfied and 0 otherwise; ${\text{rank}}_{i}$ is the rank of the first correct answer in the i-th query.

All comparative and ablation results were calculated as mean (SD) over 5 independent runs with different random initializations; the SD therefore reflects run-to-run variability. To keep the main manuscript concise, we summarized the comparative results as mean values only in the manuscript, and the full comparative mean (SD) results are provided in Tables S3-S6 in Multimedia Appendix 1. The 95% CI was calculated as mean $\pm t_{0.975,4}\times \text{SD}\text{/}\sqrt{5}$, where $t_{0.975,4}=2.776$. For each comparator, an overall *P* value was calculated using a paired *t* test over matched repeated-run results across MRR and Hits@1/3/10. In the comparative experiments, RL4TKGR served as the reference model. In the ablation experiments, the full RL4TKGR model served as the reference model. The reported *P* values are row-level overall comparisons rather than metric-specific tests.

Because MRR and Hits@k are ranking-based model metrics calculated over test queries and averaged across 5 runs, they are reported as metric values rather than participant proportions. The test query counts were 27,206 for CG-TKG-4, 4797 for CG-TKG-8, and 3031 for CG-TKG-12, as shown in Table 1.

For link prediction evaluation, we used a time-aware filtered setting rather than a raw or static filtered setting. Specifically, for each test query (s,p,…,t), candidate entities $o^{`}$ that form known true quadruples $\left(s,p,o^{`},t\right)$ with the same subject, relation, and time stamp in the training, validation, or test split were removed from the ranking list, except for the target answer. True quadruples occurring at different time stamps were retained because static filtering incorrectly removes temporally valid alternatives and is therefore unsuitable for TKG reasoning.

The maximum reasoning path length was fixed at L=3 for all experiments. This setting follows the TITer-style path-based reinforcement learning paradigm for TKG reasoning [19], in which a small fixed number of hops is used to control search noise and computational cost. It also matched the visit-level CG-TKG setting because a 3-hop trajectory can traverse the main historical diagnosis or prescription chain while avoiding unnecessarily long paths to weakly related nodes.

### Computational Cost and Convergence

All experiments were conducted on a workstation with one NVIDIA GeForce RTX 4090 GPU (24 GB), an Intel Core i7 CPU, and 16‐32 GB system RAM. With a batch size of 512, CG-TKG-4 contains approximately 316 batches per epoch and requires approximately 1.5 minutes to 3 minutes per epoch, with a total training time of approximately 2 hours to 4 hours. CG-TKG-8 contains approximately 134 batches per epoch and requires approximately 40 seconds to 80 seconds per epoch, with a total training time of approximately 1 hour to 2 hours. CG-TKG-12 contains approximately 72 batches per epoch and requires approximately 25 seconds to 45 seconds per epoch, with a total training time of approximately 0.5 hour to 1 hour. The peak GPU memory usage is approximately 2 GB to 4 GB for CG-TKG-4 and 2 GB to 3 GB for CG-TKG-8 and CG-TKG-12; peak system memory usage is approximately 4 GB to 8 GB, 3 GB to 6 GB, and 3 GB to 5 GB, respectively. Training uses a maximum of 50‐100 epochs with early stopping based on validation MRR and a patience of 5‐10 epochs. Models typically converge within approximately 40‐70 epochs, and CG-TKG-12 converges earlier than CG-TKG-4 because it contains fewer training quadruples and a more stable long-course visit structure. Based on the Table 1 test-set sizes (27,206, 4797, and 3031 queries for CG-TKG-4, CG-TKG-8, and CG-TKG-12, respectively) and an average path-based reinforcement learning inference latency of approximately 2 ms per query on the same RTX 4090 GPU, test-set inference is estimated to require approximately 54 seconds, 10 seconds, and 6 seconds, respectively. The relatively small patient count in CG-TKG-12 may increase the risk of overfitting to long-course patient trajectories. To reduce this risk, we used separate training, validation, and test splits, validation-MRR-based early stopping, 5 independent runs with different random initializations, and comparative and ablation analyses. Nevertheless, external validation on larger and more balanced cohorts is needed.

### Introduction to Baseline Models

Here, we present the baseline models used for comparison. Static knowledge graph reasoning models included TransE [14], relational graph convolutional network (R-GCN) [35], structure-aware convolutional network (SACN) [36], and DistMult [16], while the TKG reasoning models included RE-GCN [19], CEN [27], RE-Net [28], CENET [29], and TITer [26], as well as 3 recent TKG reasoning baselines: TiRGN [27], RETIA [28], and DaeMon [29]. For fairness, all baseline models were configured with their optimal hyperparameters as reported in the original papers or tuned to achieve their best performance. Detailed descriptions of the baseline models are provided in Table S1 in Multimedia Appendix 1.

## Results

### Comparative Experiments

RL4TKGR was compared with the models listed in the Baseline Models section. The static knowledge graph models were retained as conventional reference baselines, but the primary fairness comparison was based on TKG reasoning models that can use temporal information. All baseline models used optimal performance parameters. The comparative experimental results are shown in Table 2. To keep the main manuscript concise, Table 2 reports mean values only in a compact cross-dataset column format; the full comparative results with SDs and row-level paired *t* test *P* values are provided in Tables S3-S6 in Multimedia Appendix 1.

**Table 2.** Comparative experimental results.

Model	CG-TKG^a^-4				CG-TKG-8				CG-TKG-12			
	MRR^b^	Hits@1	Hits@3	Hits@10	MRR	Hits@1	Hits@3	Hits@10	MRR	Hits@1	Hits@3	Hits@10
TransE	24.34	1.17	31.13	39.41	23.60	1.89	29.45	37.59	24.92	10.44	32.36	36.08
R-GCN^c^	13.39	6.21	14.51	27.34	15.72	7.21	15.33	28.51	14.15	6.31	14.25	31.17
SACN^d^	20.28	11.80	20.99	36.95	22.91	18.76	27.13	39.35	21.89	9.93	25.27	39.08
DistMult	26.83	14.69	30.48	53.50	25.80	16.06	30.81	49.80	29.88	22.12	38.07	45.77
CEN^e^	29.55	19.51	32.88	47.69	30.40	18.94	34.49	50.06	31.19	20.91	36.71	51.02
RE-GCN^f^	30.98	21.60	34.50	49.22	31.55	20.84	35.69	51.19	32.13	23.34	38.23	53.59
RE-Net^g^	31.89	22.40	34.55	50.17	32.49	21.07	37.45	53.09	35.38	25.67	38.90	55.07
CENET^h^	35.11	29.45	50.71	59.37	37.69	30.96	50.39	60.25	40.32	31.42	52.69	63.92
TITer^i^	34.02	31.48	35.40	39.98	50.56	47.97^j^	52.64	53.93	49.55	42.05	56.57	62.36
TiRGN^k^	37.32	32.47	38.53	41.97	50.92	41.10^l^	53.12	55.93	50.51	43.31	56.82	63.15
RETIA^m^	42.12	33.83	40.65	48.43	51.14	45.57^l^	56.93	57.14	53.76	44.95	59.07	67.28
DaeMon^n^	43.26	34.41	43.90	51.54	51.76	46.60^l^	57.45	63.94	54.87	45.27	60.11	68.95
RL4TKGR^o^,^p^	51.66^j^	35.39^j^	53.14^j^	60.15^j^	53.18^j^	45.48	59.52^j^	67.26^j^	56.95^j^	49.75^j^	62.54^j^	71.79^j^

a CG-TKG: chronic gastritis temporal knowledge graph.

b MRR: mean reciprocal rank.

c R-GCN: relational graph convolutional network.

d SACN: structure-aware convolutional network.

e CEN: complex evolutional network.

f RE-GCN: relation-enhanced graph convolutional network.

g RE-Net: recurrent event network.

h CENET: contrastive event network.

i TITer: TimeTraveler.

j Best result in the column.

k TiGRN: time-guided recurrent graph network.

l Second-best result in the column.

m RETIA: relation-entity twin-interact aggregation.

n DaeMon: adaptive path-memory network.

o Reference model.

p RL4TKGR: reinforcement learning–based temporal knowledge graph reasoning

Table 2 shows the comparison of prediction performance between RL4TKGR and various static knowledge graph reasoning models and TKG reasoning models on the 3 temporal datasets (CG-TKG-4/8/12). H1, H3, and H10 denote Hits@1, Hits@3, and Hits@10, respectively. The results from the overall paired *t* tests across MRR and Hits@1/3/10 were significantly different between RL4TKGR and all baseline models on CG-TKG-4 and CG-TKG-12 (*P*<.001 for all comparisons). For CG-TKG-8, the differences were also significant for all baselines (*P*<.001), except for TITer, for which the overall paired *t* test yielded a *P*=.004. For the 3 newly recent baselines, the comparisons between RL4TKGR and TiRGN, RETIA, and DaeMon all yielded *P*<.001 on CG-TKG-4, CG-TKG-8, and CG-TKG-12. These results indicate that temporal modeling is important for analyzing the evolutionary process of chronic gastritis, while the remaining Hits@1 advantage of TITer on CG-TKG-8 is reported as a metric-specific exception rather than as evidence of a specific mechanism.

RL4TKGR achieved the highest MRR, Hits@3, and Hits@10 on all 3 datasets and the highest Hits@1 on CG-TKG-4 and CG-TKG-12. DaeMon had the strongest MRR at baseline on all 3 subsets. Compared with DaeMon, RL4TKGR increased MRR from 43.26 (SD 0.95; 95% CI 42.08‐44.44) to 51.66 (SD 0.76; 95% CI 50.72‐52.60) on CG-TKG-4, from 51.76 (SD 1.12; 95% CI 50.37‐53.15) to 53.18 (SD 0.79; 95% CI 52.20‐54.16) on CG-TKG-8, and from 54.87 (SD 1.17; 95% CI 53.42‐56.32) to 56.95 (SD 0.84; 95% CI 55.91‐57.99) on CG-TKG-12. For Hits@10, RL4TKGR also outperformed DaeMon by reaching MRRs of 60.15 (SD 0.91) versus 51.54 (SD 1.08) on CG-TKG-4, 67.26 (SD 0.96) versus 63.94 (SD 1.31) on CG-TKG-8, and 71.79 (SD 1.03) versus 68.95 (SD 1.39) on CG-TKG-12. The only first-rank exception was Hits@1 on CG-TKG-8, where TITer reached 47.97 (SD 1.18), DaeMon reached 46.60 (SD 1.04), and RL4TKGR reached 45.48 (SD 0.71). Because Hits@1 requires the correct answer to be ranked first, we interpreted this as a metric-specific exception rather than as evidence that RL4TKGR was uniformly superior on every ranking criterion. Overall, however, the updated comparison indicated that RL4TKGR retains a consistent advantage over recent TKG reasoning models in MRR, Hits@3, and Hits@10, while the static baselines were retained only as conventional reference models.

### Ablation Study

Ablation experiments were designed to verify the effectiveness of the historical diagnosis encoding module and historical reward in RL4TKGR. The experiments removed the historical diagnosis encoding module (w/o e), removed the historical reward (w/o r), removed both simultaneously (w/o e&r), and evaluated their performance on the 3 datasets CG-TKG-4, CG-TKG-8, and CG-TKG-12. Table 3 reports the experimental results as mean (SD) over 5 runs. The full model resulted in significant overall differences from all ablated versions in paired *t* tests across MRR and Hits@1/3/10 (*P*<.001 for all comparisons).

**Table 3.** Ablation study results.

Model	CG-TKG^a^-4				CG-TKG-8				CG-TKG-12			
	MRR^b^	Hits@1	Hits@3	Hits@10	MRR	Hits@1	Hits@3	Hits@10	MRR	Hits@1	Hits@3	Hits@10
w/o e^c^	44.05	33.85	47.65	55.34	50.53	41.91	55.95	64.86	54.30	46.43	63.54^d^	68.32
w/o r^e^	41.59	30.18	45.93	52.18	49.55	40.04	54.56	62.35	53.16	44.72	60.88	66.39
w/o e&r^f^	33.54	28.75	39.12	45.89	34.49	31.52	36.56	39.52	42.95	36.51	44.76	57.66
RL4TKGR^g^	51.66^d^	35.39^d^	53.14^d^	60.15^d^	53.18^d^	45.48^d^	59.52^d^	67.26^d^	56.95^d^	49.75^d^	62.54	71.79^d^

a CG-TKG: chronic gastritis temporal knowledge graph.

b MRR: mean reciprocal rank.

c w/o e: historical diagnosis encoding module removed.

d Best result in the column.

e w/o r historical reward removed.

f w/o e&r: historical diagnosis encoding module and historical rewarad simultaneously removed.

g RL4TKGR: reinforcement learning–based temporal knowledge graph reasoning.

Specifically, on the CG-TKG-4 dataset, the MRR of the full model was 51.66 (SD 0.76) compared with 44.05 (SD 0.82) for w/o e, 41.59 (SD 0.88) for w/o r, and 33.54 (SD 0.74) for w/o e&r. This pattern was also observed on CG-TKG-8 and CG-TKG-12 for MRR, Hits@1, and Hits@10. For Hits@3 on CG-TKG-12, however, w/o e reached an MRR of 63.54 (SD 0.98), which was higher than the full model value of 62.54 (SD 0.93). This exception indicated that the historical diagnosis encoding module does not monotonically improve every individual ranking metric. Nevertheless, the full RL4TKGR model retained higher MRR, Hits@1, and Hits@10 on CG-TKG-12 and had the best overall ablation profile across the 3 datasets.

Comparing the impact of ablating a single module, the removal of the historical diagnosis encoding module (w/o e) caused slightly more damage to performance than the removal of the historical reward (w/o r). This indicated that the diagnosis encoding module played a more fundamental role in modeling patients’ historical states. Nevertheless, when both modules were ablated simultaneously (w/o e&r), the performance exhibited a steep decline, which suggested that the historical reward substantially enhanced the effectiveness of the diagnosis encoding module and that the 2 components demonstrated a strong complementary relationship.

### Experiments on the Effectiveness of the Event Balancing Factor in the Reward Function

**Figure 2.**
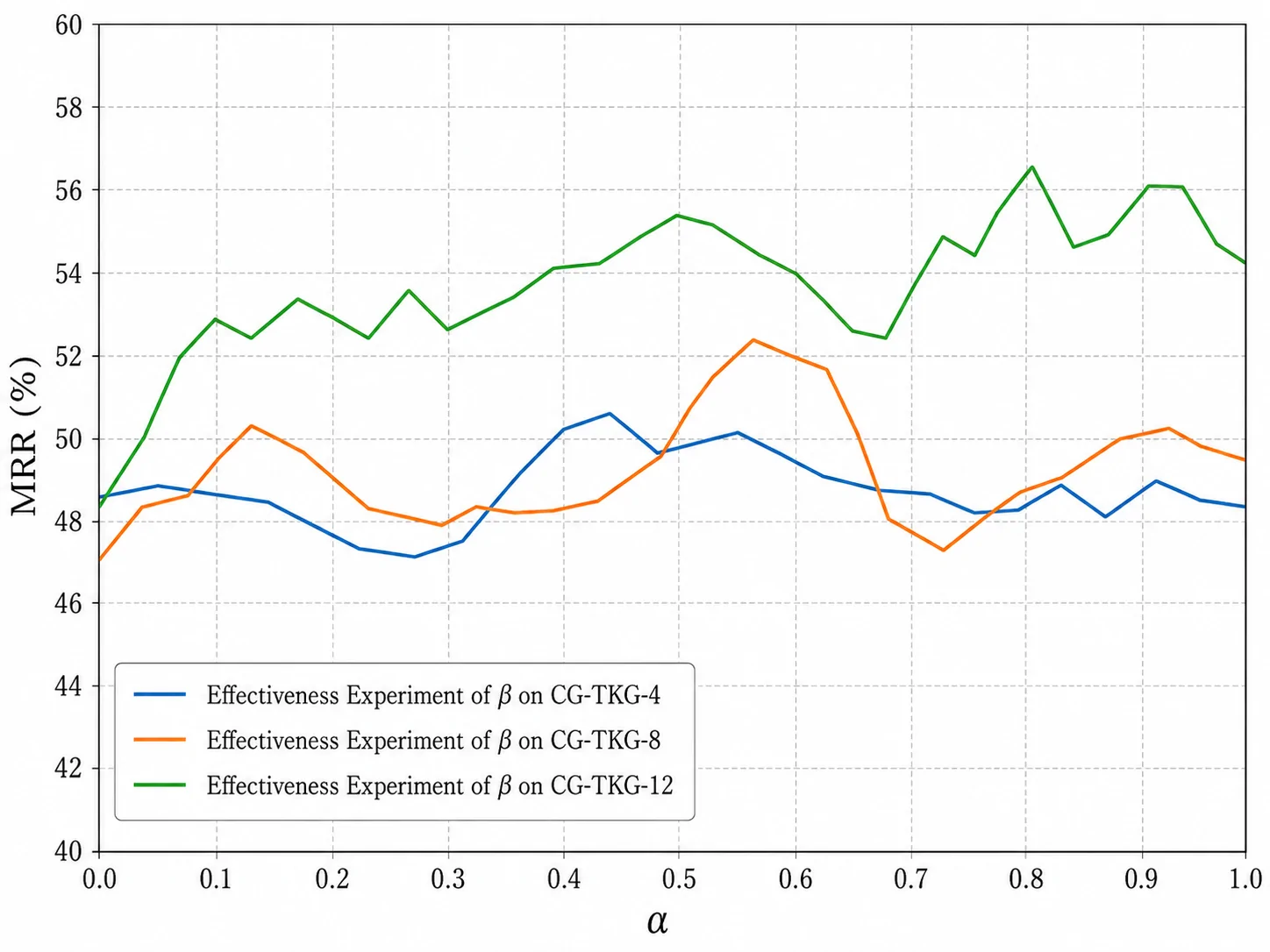
Effectiveness experiment of β on chronic gastritis temporal knowledge graph (CG-TKG)-4, CG-TKG-8, and CG-TKG-12. MRR: mean reciprocal rank.

In the RL4TKGR model, the reward function calculated the weighted value of the historical reward and the nonhistorical reward using a hyperparameter α, where α was provided by the value of the event balance factor β. To verify the impact of the event balance factor β in the reward function on model performance, experiments were conducted on 3 different datasets: CG-TKG-4, CG-TKG-8, and CG-TKG-12. The hyperparameter α in the reward function was fixed to guide the learning of the policy network, and a hyperparameter search was performed on each dataset with α ranging from 0 to 1. The experimental results are shown in Figure 2, where the 3 curves represent the fixed-α MRR sensitivity results on CG-TKG-4, CG-TKG-8, and CG-TKG-12, respectively. On CG-TKG-4, when α was fixed, the MRR value ranged from 45.0 to 51.0, while the MRR value of RL4TKGR was 51.66. On CG-TKG-8 and CG-TKG-12, the best fixed-α settings appeared around 0.55 and 0.8, respectively. These descriptive trends suggested that longer visit histories placed greater weight on historical diagnostic information. Because no paired *t* test *P* value was calculated for the fixed-α hyperparameter sweep, these results are reported as descriptive sensitivity analyses rather than formal significance tests.

In summary, the application of the event balance factor β in the reward function had a consistent descriptive effect on model performance. In particular, when trained jointly with the policy network, β could adaptively adjust the weights of historical and nonhistorical information, thereby optimizing the overall performance of the model. Therefore, dynamically adjusting β was an effective means of improving model performance.

## Discussion

### Model Interpretability Analysis

In the dynamic weight allocator module, the event balance factor β dynamically and interpretably adjusted the weight distribution between the dual-channel aggregation reward function and the action scoring module. When the query focused on historical diagnosis information, β approached 1; when the query focused on nonhistorical diagnosis information, β approaches 0.

The factor β enabled the model to automatically and interpretably adjust the source weights of decision-making bases according to the patient’s specific current condition. In the design of the dual-channel aggregation reward function, the value of α was directly provided by β. As shown in the hyperparameter experimental results in Figure 2, the interpretability was explained as follows: (1) On CG-TKG-4, in which the maximum number of patient visits was 4, the probability of disease progression was relatively high, whereas historical correlations were weak. The model attained its highest MRR when α was between 0.4 and 0.5, providing evidence of its interpretability. (2) On CG-TKG-8, in which the maximum number of patient visits was 8, the disease state tended to stabilize, the likelihood of progression decreased, and historical correlations became stronger. The model achieved its highest MRR when α was between 0.5 and 0.6. (3) On CG-TKG-12, where the maximum number of patient visits was twelve, the disease state was the most stable, the likelihood of progression was the lowest, and historical correlations were the strongest. The model attained its highest MRR when α is approximately 0.8.

**Figure 3.**
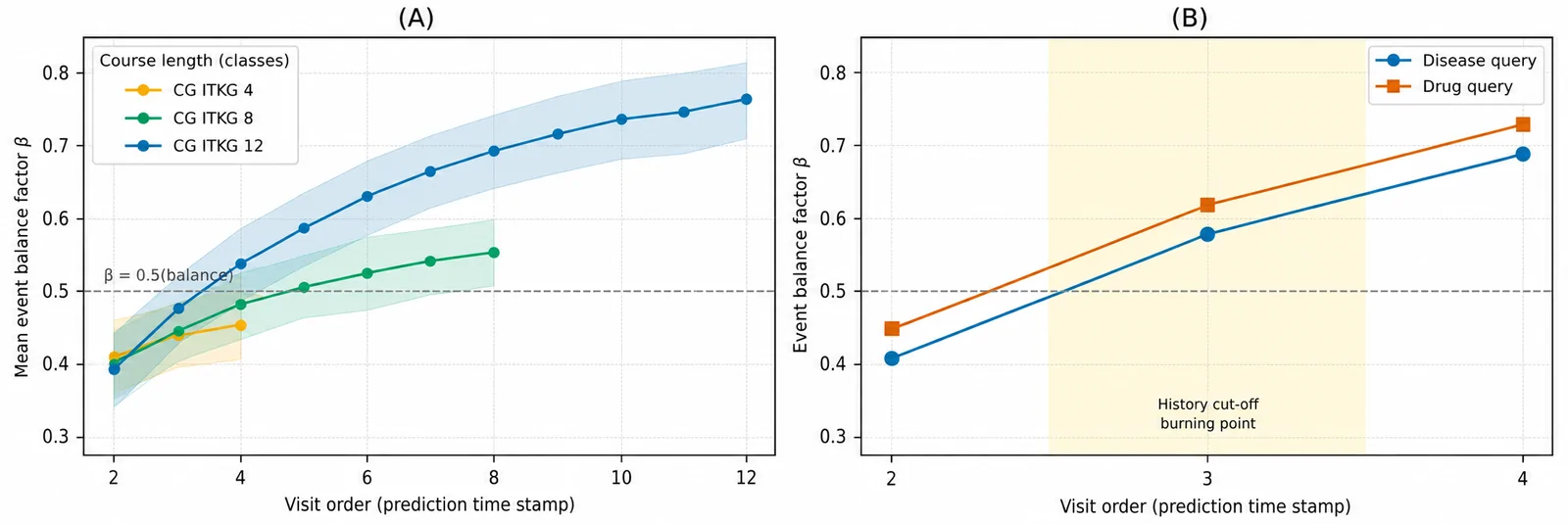
Patient-level interpretability of the event balance factor β: (A) mean (SD in shaded areas) query-level β values across visit order for chronic gastritis temporal knowledge graph (CG-TKG)-4, CG-TKG-8, and CG-TKG-12 and (B) β trajectory of the illustrative anonymized case (Patient A) for disease and medication prediction queries. Values >0.5 indicate history-weighted reasoning, whereas values <0.5 indicate greater reliance on nonhistorical or current-visit information.

To further examine patient-level interpretability, we analyzed 1686 query-level β values exported during model inference. As shown in Figure 3, the mean β increased with visit order across all 3 datasets, from 0.413 to 0.462 in CG-TKG-4, from 0.400 to 0.557 in CG-TKG-8, and from 0.393 to 0.763 in CG-TKG-12. Because larger β values indicate greater reliance on the historical diagnostic path, this pattern indicated that RL4TKGR progressively increased the weight assigned to accumulated historical information as longitudinal visit information became richer. In the illustrative anonymized case, Patient A had the same direction of change: β increased from 0.41 to 0.69 for disease prediction and from 0.45 to 0.73 for medication prediction between visits 2 and 4. These patient-level trajectories provided a direct visualization of how the model dynamically shifted from nonhistorical or current visit information toward history-weighted reasoning.

### Path-Level and Counterfactual Interpretability

The event balance factor β should not be interpreted as a conventional clinical attention weight. Instead, it is a query-level model weight that quantifies how strongly the policy relies on historical versus nonhistorical reasoning paths. To move beyond a single top-ranked case result, we exported the actual reasoning paths for Patient A and provide them in Tables S13-S15 in Multimedia Appendix 1. For the disease diagnosis query at the third visit, RL4TKGR predicted chronic gastritis with β=0.58 and followed the historical diagnostic chain Patient A → chronic gastritis at visit 1 → chronic gastritis at visit 2 → the third-visit diagnosis query. For the medication query, RL4TKGR predicted Zhizhu Kuanzhong capsules with β=0.62 and followed the previous prescription path across the first 2 visits.

The same exported paths also showed the nonhistorical component of the prediction. For the disease diagnosis query, the current visit symptom path linked abdominal distension, poor belching, and phlegm in the pharynx to chronic gastritis, with a nonhistorical contribution of 1−β=0.42. For the medication query, the current visit symptom path linked abdominal distension and loose stools to Zhizhu Kuanzhong capsules, with a nonhistorical contribution of 1−β=0.38. As a patient-level counterfactual check, when the historical path was masked by forcing β=0, the true disease diagnosis fell from rank 1 to rank 4, and the true medication fell from rank 1 to rank 5. These results indicated that the historical reasoning path contributed to the rank-1 predictions rather than merely accompanying them.

At the cohort level, the ablation study provided an additional counterfactual view of interpretability. Removing the historical diagnosis encoding module or the historical reward substantially lowered MRR across the CG-TKG datasets, indicating that the historical path components were not decorative but materially contributed to model performance. Thus, the interpretability evidence in RL4TKGR consisted of query-level β weighting, exported path-level evidence, and counterfactual changes after masking or removing historical-path information.

### Case Analysis of Chronic Gastritis Diagnosis Prediction

We selected 3 visits by an anonymized case (Patient A) as examples for analysis. Patient A was a study-specific anonymous label and did not correspond to any original hospital identifier. To reduce the risk of reidentification, the case is reported using visit order rather than calendar dates. This case corresponds to the patient-level β trajectory shown in Figure 3B, providing a link between the model’s dynamic weighting mechanism and the case-level prediction results provided in the following paragraphs.

In the first visit, the manifestations included occasional stomach pain after acute gastroenteritis, abdominal distension, poor belching, a foreign body sensation in the pharynx, phlegm in the pharynx, normal appetite, average sleep quality with many dreams, and normal urination and defecation. The tongue was dark red with a thin white coating, and the pulse was deep and thready. The diagnosis was chronic gastritis, and the prescribed medicines were Zhizhu Kuanzhong capsules and Qingyan tablets.

In the second visit, the manifestations were disappearance of stomach pain, occasional abdominal distension, a foreign body sensation in the pharynx, poor belching, phlegm in the pharynx, normal appetite, average sleep quality, improvement in dreaminess, and normal urination and defecation. The tongue was dark red with a thin white coating that was slightly yellowish, and the pulse was deep and thready. The diagnosis was chronic gastritis, and the prescribed medicines were Zhizhu Kuanzhong capsules and Qingyan Tablets.

In the third visit, the manifestations were loose stools in the past week, occasional abdominal distension, poor belching, phlegm in the pharynx, normal appetite, average sleep quality with many dreams, and normal urination. The tongue was dark red with a thin white coating, and the pulse was deep and thready.

Using RL4TKGR, based on the historical diagnostic information for Patient A and the symptom information from the third visit, the top 10 probabilistic reasoning results for the anonymized third-visit queries (Patient A, prescribe medicine, ?, 3) and (Patient A, disease diagnosis, ?, 3) were generated. As shown in Table 4, the actual results from the third visit for Patient A are shown among the other results. The analysis showed that, in both query cases, RL4TKGR ranked the correct result first in the prediction sequence, suggesting that the model could rank plausible candidate diagnoses and medications for clinician review while preserving links to temporal medical entities.

**Table 4.** Case reasoning results.

Ranking	(Patient 49375, prescribe medicine, ?, 3), predicted entities	(Patient 49375, disease diagnosis, ?, 3), predicted entities
1	Zhizhu Kuanzhong capsules^a^	Chronic gastritis**^a^**
2	Guben Yichang tablets^a^	Chronic atrophic gastritis
3	Simo Decoction oral liquid	Chronic superficial gastritis
4	Bazhen Yimu pills	Erosive gastritis
5	Qingyan tablets	*Helicobacter pylori*–associated gastritis
6	Xuhanting capsules	Spleen deficiency and dampness-heat syndrome
7	Wuweizi granules	Mild dysplasia of chronic gastritis
8	Qizhi Weitong granules	Chronic gastritis with intestinal metaplasia
9	Xuhan Weitong granules	Chronic gastritis with hiatal hernia
10	Jinghua Weikang capsules	Liver stagnation and spleen deficiency syndrome

a Actual results from the third visit for Patient A.

The subsequent follow-up results for Patient A, represented as the fourth visit in the anonymized sequence, indicated marked clinical improvement: alleviated abdominal distension, belching, reduced night sweats, general chills, disappearance of phlegm, normal appetite, average sleep, sometimes formed stools, and normal urination. The patient was diagnosed with chronic gastritis. The patient’s reported symptoms improved after taking the prescribed drugs, and the further progression of chronic gastritis was controlled in this illustrative case. These results supported the plausibility of the predicted medication ranking and indicated that RL4TKGR may help predict future disease status and recommend candidate therapies for clinical review.

### Conclusion

We investigated the temporal dependency of chronic gastritis progression and, based on CG-TKG constructed from TCM diagnostic and treatment data, proposed RL4TKGR, a reinforcement learning–based TKG reasoning model. By designing a reinforcement learning policy network that exploits the associations among historical diagnosis data, the model achieved interpretable prediction of chronic gastritis diagnosis and treatment. The experimental results support the effectiveness of RL4TKGR on the evaluated CG-TKG reasoning tasks.

RL4TKGR may provide model-level support for ranking candidate diagnoses and medications in longitudinal chronic gastritis care. Because the current CG-TKG is constructed from a TCM-specific symbolic entity space and has not been externally validated across datasets, institutions, or disease domains, its generalizability to other clinical settings remains to be established. In addition, because the data originate from one clinical setting and one chief physician’s practice, the model’s performance may not directly generalize to other hospitals, clinicians, regional practice styles, or patient populations without external validation. Future work should first evaluate whether the architecture can be adapted to other longitudinal clinical knowledge graphs through disease-specific schema design, external validation, and prospective clinical evaluation. From a translational perspective, RL4TKGR may serve as a prototype decision support tool that ranks candidate diagnoses and medications for clinician review in longitudinal chronic gastritis care. Future prospective validation and collaboration among clinicians, data governance teams, and machine learning researchers are needed before deployment in real-world clinical workflows.

Another limitation is that the current CG-TKG uses visit-order time stamps rather than continuous calendar time. Although Table 1 reports the mean and variance of visit intervals, RL4TKGR does not explicitly use elapsed days between visits. Future work should incorporate time-gap embeddings, temporal decay functions, or continuous-time TKG models to capture heterogeneous follow-up intervals more precisely.

## Supplementary material

10.2196/83544 Multimedia Appendix 1 Baseline model descriptions, evaluation metrics and statistical reporting, full comparative results, full ablation results, fixed-alpha sensitivity analysis results, extended case analysis, model construction and quality control, statistical CIs and computational cost, specific case findings, terminology glossary, and model training and inference pseudocode.

## Abbreviations

AI: artificial intelligence
CEN: complex evolutional network
CENET: contrastive event network
CG-TKG: chronic gastritis temporal knowledge graph
DaeMon: adaptive path-memory network
EHR: electronic health record
EMR: electronic medical record
GRU: gated recurrent unit
LSTM: long short-term memory
MLP: multilayer perceptron
MRR: mean reciprocal rank
R-GCN: relational graph convolutional network
RE-GCN: relation-enhanced graph convolutional network
RE-Net: recurrent event network
RETIA: relation-entity twin-interact aggregation
RL4TKGR: reinforcement learning–based temporal knowledge graph reasoning
RNN: recurrent neural network
RTTI: run-time type information
SACN: structure-aware convolutional network
TANGO: Temporal Knowledge Graph Forecasting with Neural Ordinary Equations
TCM: traditional Chinese medicine
TiGRN: time-guided recurrent graph network
TITer: TimeTraveler
TKG: temporal knowledge graph
