# Supplementary material for: Reinforcement Learning–Based Temporal Knowledge Graph Reasoning for Predicting Chronic Gastritis Diagnosis and Treatment: Development and Validation Study
Source: JMIR Med Inform. 2026 Sep 11;14:e83544. doi: 10.2196/83544 (PMC13578680; doi:10.2196/83544)
Supplement: Supplementary file 1 [file medinform-v14-e83544-s001.docx]

# 1. Baseline Model Descriptions

The main manuscript summarizes the baseline families. The expanded descriptions below document the comparator models used in the comparative experiments. Full citations are provided in the main manuscript reference list.

Table S1. Baseline models used for comparison.

| Model | Supplementary description |
| --- | --- |
| TransE | Models relations as translation operations in the embedding space, using h + r approximately equals t for each triple. It is parameter efficient and scalable for large knowledge bases. |
| R-GCN | Extends graph convolutional networks to multi-relational data by assigning relation-specific transformation matrices and aggregating neighborhood features. |
| SACN | Combines a weighted graph convolutional network with a Conv-TransE decoder to model relation-aware edge importance and triple-level semantic constraints. |
| DistMult | Represents each relation as a diagonal matrix. It is efficient for symmetric relations but is limited for asymmetric relations. |
| RE-GCN | Integrates graph convolution, recurrent gating, and static constraints to model entity evolution and structural importance over time. |
| CEN | Learns complex temporal evolution patterns by decoupling periodic and aperiodic patterns and reusing historical states through time-aware replication. |
| RE-Net | Uses a recurrent temporal encoder and a global neighborhood aggregator to model historical event sequences and concurrent events at the same timestamp. |
| CeNet | Uses historical contrastive learning to identify key historical events and an entity drift module to address representation changes across time. |
| TiTer | Introduces reinforcement learning for temporal knowledge graph reasoning by formulating time-aware path search as a Markov decision process. |
| TiRGN | Time-guided recurrent graph network with local-global historical patterns for temporal knowledge graph reasoning. |
| RETIA | Relation-entity twin-interact aggregation model for temporal knowledge graph extrapolation, designed to jointly model relation and entity interactions. |
| DaeMon | Adaptive path-memory network that captures historical path information for temporal knowledge graph reasoning. |

# 2. Evaluation Metrics and Statistical Reporting

Model performance was evaluated using mean reciprocal rank (MRR) and Hits@k. Results are reported on the same scale as the main manuscript. Comparative and ablation results are presented as mean (SD) over five runs. Primary MRR confidence intervals and computational-cost details are provided in Section 8.

Table S2. Evaluation metric definitions.

| Metric | Formula | Interpretation |
| --- | --- | --- |
| MRR | MRR = (1 / \|Q\|) sum_i 1 / rank_i | Average reciprocal rank of the first correct answer across all queries. |
| Hits@k | Hits@k = (1 / \|Q\|) sum_i I(rank_i <= k) | Proportion of queries for which the correct answer appears in the top k ranked candidates. |

Statistical note: For each comparator, the P value is an overall paired t test across MRR, Hits@1, Hits@3, and Hits@10. In comparative experiments, RL4TKGR is the reference model. In ablation experiments, the full RL4TKGR model is the reference model. P values are not metric-specific. The 95% CI is calculated as mean +/- t0.975,4 x SD / sqrt(5), with t0.975,4 = 2.776.

# 3. Full Comparative Results

The following tables provide the full comparative results for the three CG-TKG subsets. They correspond to the summarized comparison reported in the main manuscript. To reduce the length of the main manuscript, Table 2 now reports mean values only, whereas this section provides the full mean (SD) comparative results and row-level paired-test P values.

Table S3. Full comparative results corresponding to main Table 2, reported as mean (SD) over five runs.

| Dataset | Model | MRR, mean (SD) | Hits@1, mean (SD) | Hits@3, mean (SD) | Hits@10, mean (SD) |
| --- | --- | --- | --- | --- | --- |
| CG-TKG-4 | TransE | 24.34 (0.42) | 1.17 (0.09) | 31.13 (0.51) | 39.41 (0.66) |
| CG-TKG-4 | R-GCN | 13.39 (0.36) | 6.21 (0.18) | 14.51 (0.42) | 27.34 (0.58) |
| CG-TKG-4 | SACN | 20.28 (0.47) | 11.80 (0.31) | 20.99 (0.54) | 36.95 (0.71) |
| CG-TKG-4 | DistMult | 26.83 (0.58) | 14.69 (0.44) | 30.48 (0.63) | 53.50 (0.82) |
| CG-TKG-4 | CEN | 29.55 (0.61) | 19.51 (0.52) | 32.88 (0.67) | 47.69 (0.79) |
| CG-TKG-4 | RE-GCN | 30.98 (0.69) | 21.60 (0.55) | 34.50 (0.73) | 49.22 (0.85) |
| CG-TKG-4 | RE-Net | 31.89 (0.72) | 22.40 (0.61) | 34.55 (0.76) | 50.17 (0.88) |
| CG-TKG-4 | CeNet | 35.11 (0.83) | 29.45 (0.69) | 50.71 (0.94) | 59.37 (1.02) |
| CG-TKG-4 | TiTer | 34.02 (1.15) | 31.48 (1.08) | 35.40 (1.24) | 39.98 (1.31) |
| CG-TKG-4 | TiRGN | 37.32 (0.85) | 32.47 (0.78) | 38.53 (0.91) | 41.97 (0.97) |
| CG-TKG-4 | RETIA | 42.12 (0.92) | 33.83 (0.81) | 40.65 (0.95) | 48.43 (1.04) |
| CG-TKG-4 | DaeMon | 43.26 (0.95) | 34.41 (0.83) | 43.90 (0.99) | 51.54 (1.08) |
| CG-TKG-4 | **RL4TKGR** | 51.66 (0.76) | 35.39 (0.64) | 53.14 (0.82) | 60.15 (0.91) |
| CG-TKG-8 | TransE | 23.60 (0.45) | 1.89 (0.11) | 29.45 (0.49) | 37.59 (0.64) |
| CG-TKG-8 | R-GCN | 15.72 (0.39) | 7.21 (0.21) | 15.33 (0.43) | 28.51 (0.56) |
| CG-TKG-8 | SACN | 22.91 (0.51) | 18.76 (0.47) | 27.13 (0.58) | 39.35 (0.72) |
| CG-TKG-8 | DistMult | 25.80 (0.56) | 16.06 (0.42) | 30.81 (0.66) | 49.80 (0.86) |
| CG-TKG-8 | CEN | 30.40 (0.62) | 18.94 (0.55) | 34.49 (0.71) | 50.06 (0.84) |
| CG-TKG-8 | RE-GCN | 31.55 (0.70) | 20.84 (0.59) | 35.69 (0.78) | 51.19 (0.88) |
| CG-TKG-8 | RE-Net | 32.49 (0.73) | 21.07 (0.62) | 37.45 (0.81) | 53.09 (0.92) |
| CG-TKG-8 | CeNet | 37.69 (0.86) | 30.96 (0.76) | 50.39 (0.97) | 60.25 (1.05) |
| CG-TKG-8 | TiTer | 50.56 (1.21) | 47.97 (1.18) | 52.64 (1.29) | 53.93 (1.37) |
| CG-TKG-8 | TiRGN | 50.92 (1.08) | 41.10 (0.97) | 53.12 (1.15) | 55.93 (1.22) |
| CG-TKG-8 | RETIA | 51.14 (1.10) | 45.57 (1.02) | 56.93 (1.19) | 57.14 (1.25) |
| CG-TKG-8 | DaeMon | 51.76 (1.12) | 46.60 (1.04) | 57.45 (1.21) | 63.94 (1.31) |
| CG-TKG-8 | **RL4TKGR** | 53.18 (0.79) | 45.48 (0.71) | 59.52 (0.88) | 67.26 (0.96) |
| CG-TKG-12 | TransE | 24.92 (0.48) | 10.44 (0.34) | 32.36 (0.57) | 36.08 (0.69) |
| CG-TKG-12 | R-GCN | 14.15 (0.41) | 6.31 (0.24) | 14.25 (0.46) | 31.17 (0.62) |
| CG-TKG-12 | SACN | 21.89 (0.50) | 9.93 (0.35) | 25.27 (0.61) | 39.08 (0.74) |
| CG-TKG-12 | DistMult | 29.88 (0.64) | 22.12 (0.58) | 38.07 (0.76) | 45.77 (0.88) |
| CG-TKG-12 | CEN | 31.19 (0.67) | 20.91 (0.60) | 36.71 (0.79) | 51.02 (0.93) |
| CG-TKG-12 | RE-GCN | 32.13 (0.73) | 23.34 (0.66) | 38.23 (0.85) | 53.59 (0.96) |
| CG-TKG-12 | RE-Net | 35.38 (0.78) | 25.67 (0.72) | 38.90 (0.89) | 55.07 (1.01) |
| CG-TKG-12 | CeNet | 40.32 (0.91) | 31.42 (0.82) | 52.69 (1.02) | 63.92 (1.11) |
| CG-TKG-12 | TiTer | 49.55 (1.26) | 42.05 (1.15) | 56.57 (1.33) | 62.36 (1.42) |
| CG-TKG-12 | TiRGN | 50.51 (1.07) | 43.31 (0.99) | 56.82 (1.18) | 63.15 (1.29) |
| CG-TKG-12 | RETIA | 53.76 (1.14) | 44.95 (1.03) | 59.07 (1.23) | 67.28 (1.36) |
| CG-TKG-12 | DaeMon | 54.87 (1.17) | 45.27 (1.05) | 60.11 (1.26) | 68.95 (1.39) |
| CG-TKG-12 | **RL4TKGR** | 56.95 (0.84) | 49.75 (0.77) | 62.54 (0.93) | 71.79 (1.03) |

Table S4. Comparative results on CG-TKG-4, including SD and P values.

| Model | MRR, mean (SD) | Hits@1, mean (SD) | Hits@3, mean (SD) | Hits@10, mean (SD) | P value |
| --- | --- | --- | --- | --- | --- |
| TransE | 24.34 (0.42) | 1.17 (0.09) | 31.13 (0.51) | 39.41 (0.66) | P<.001 |
| R-GCN | 13.39 (0.36) | 6.21 (0.18) | 14.51 (0.42) | 27.34 (0.58) | P<.001 |
| SACN | 20.28 (0.47) | 11.80 (0.31) | 20.99 (0.54) | 36.95 (0.71) | P<.001 |
| DistMult | 26.83 (0.58) | 14.69 (0.44) | 30.48 (0.63) | 53.50 (0.82) | P<.001 |
| CEN | 29.55 (0.61) | 19.51 (0.52) | 32.88 (0.67) | 47.69 (0.79) | P<.001 |
| RE-GCN | 30.98 (0.69) | 21.60 (0.55) | 34.50 (0.73) | 49.22 (0.85) | P<.001 |
| RE-Net | 31.89 (0.72) | 22.40 (0.61) | 34.55 (0.76) | 50.17 (0.88) | P<.001 |
| CeNet | 35.11 (0.83) | 29.45 (0.69) | 50.71 (0.94) | 59.37 (1.02) | P<.001 |
| TiTer | 34.02 (1.15) | 31.48 (1.08) | 35.40 (1.24) | 39.98 (1.31) | P<.001 |
| TiRGN | 37.32 (0.85) | 32.47 (0.78) | 38.53 (0.91) | 41.97 (0.97) | P<.001 |
| RETIA | 42.12 (0.92) | 33.83 (0.81) | 40.65 (0.95) | 48.43 (1.04) | P<.001 |
| DaeMon | 43.26 (0.95) | 34.41 (0.83) | 43.90 (0.99) | 51.54 (1.08) | P<.001 |
| RL4TKGR | 51.66 (0.76) | 35.39 (0.64) | 53.14 (0.82) | 60.15 (0.91) | Reference |

Table S5. Comparative results on CG-TKG-8, including SD and P values.

| Model | MRR, mean (SD) | Hits@1, mean (SD) | Hits@3, mean (SD) | Hits@10, mean (SD) | P value |
| --- | --- | --- | --- | --- | --- |
| TransE | 23.60 (0.45) | 1.89 (0.11) | 29.45 (0.49) | 37.59 (0.64) | P<.001 |
| R-GCN | 15.72 (0.39) | 7.21 (0.21) | 15.33 (0.43) | 28.51 (0.56) | P<.001 |
| SACN | 22.91 (0.51) | 18.76 (0.47) | 27.13 (0.58) | 39.35 (0.72) | P<.001 |
| DistMult | 25.80 (0.56) | 16.06 (0.42) | 30.81 (0.66) | 49.80 (0.86) | P<.001 |
| CEN | 30.40 (0.62) | 18.94 (0.55) | 34.49 (0.71) | 50.06 (0.84) | P<.001 |
| RE-GCN | 31.55 (0.70) | 20.84 (0.59) | 35.69 (0.78) | 51.19 (0.88) | P<.001 |
| RE-Net | 32.49 (0.73) | 21.07 (0.62) | 37.45 (0.81) | 53.09 (0.92) | P<.001 |
| CeNet | 37.69 (0.86) | 30.96 (0.76) | 50.39 (0.97) | 60.25 (1.05) | P<.001 |
| TiTer | 50.56 (1.21) | 47.97 (1.18) | 52.64 (1.29) | 53.93 (1.37) | P=.004 |
| TiRGN | 50.92 (1.08) | 41.10 (0.97) | 53.12 (1.15) | 55.93 (1.22) | P<.001 |
| RETIA | 51.14 (1.10) | 45.57 (1.02) | 56.93 (1.19) | 57.14 (1.25) | P<.001 |
| DaeMon | 51.76 (1.12) | 46.60 (1.04) | 57.45 (1.21) | 63.94 (1.31) | P<.001 |
| RL4TKGR | 53.18 (0.79) | 45.48 (0.71) | 59.52 (0.88) | 67.26 (0.96) | Reference |

Table S6. Comparative results on CG-TKG-12, including SD and P values.

| Model | MRR, mean (SD) | Hits@1, mean (SD) | Hits@3, mean (SD) | Hits@10, mean (SD) | P value |
| --- | --- | --- | --- | --- | --- |
| TransE | 24.92 (0.48) | 10.44 (0.34) | 32.36 (0.57) | 36.08 (0.69) | P<.001 |
| R-GCN | 14.15 (0.41) | 6.31 (0.24) | 14.25 (0.46) | 31.17 (0.62) | P<.001 |
| SACN | 21.89 (0.50) | 9.93 (0.35) | 25.27 (0.61) | 39.08 (0.74) | P<.001 |
| DistMult | 29.88 (0.64) | 22.12 (0.58) | 38.07 (0.76) | 45.77 (0.88) | P<.001 |
| CEN | 31.19 (0.67) | 20.91 (0.60) | 36.71 (0.79) | 51.02 (0.93) | P<.001 |
| RE-GCN | 32.13 (0.73) | 23.34 (0.66) | 38.23 (0.85) | 53.59 (0.96) | P<.001 |
| RE-Net | 35.38 (0.78) | 25.67 (0.72) | 38.90 (0.89) | 55.07 (1.01) | P<.001 |
| CeNet | 40.32 (0.91) | 31.42 (0.82) | 52.69 (1.02) | 63.92 (1.11) | P<.001 |
| TiTer | 49.55 (1.26) | 42.05 (1.15) | 56.57 (1.33) | 62.36 (1.42) | P<.001 |
| TiRGN | 50.51 (1.07) | 43.31 (0.99) | 56.82 (1.18) | 63.15 (1.29) | P<.001 |
| RETIA | 53.76 (1.14) | 44.95 (1.03) | 59.07 (1.23) | 67.28 (1.36) | P<.001 |
| DaeMon | 54.87 (1.17) | 45.27 (1.05) | 60.11 (1.26) | 68.95 (1.39) | P<.001 |
| RL4TKGR | 56.95 (0.84) | 49.75 (0.77) | 62.54 (0.93) | 71.79 (1.03) | Reference |

# 4. Full Ablation Results

The ablation experiments removed the historical diagnosis encoding module (w/o e), the historical reward (w/o r), and both components simultaneously (w/o e&r).

Table S7. Ablation results on CG-TKG-4.

| Variant | MRR, mean (SD) | Hits@1, mean (SD) | Hits@3, mean (SD) | Hits@10, mean (SD) | P value |
| --- | --- | --- | --- | --- | --- |
| w/o e | 44.05 (0.82) | 33.85 (0.69) | 47.65 (0.91) | 55.34 (1.02) | P<.001 |
| w/o r | 41.59 (0.88) | 30.18 (0.73) | 45.93 (0.94) | 52.18 (1.07) | P<.001 |
| w/o e&r | 33.54 (0.74) | 28.75 (0.68) | 39.12 (0.83) | 45.89 (0.95) | P<.001 |
| RL4TKGR (full) | 51.66 (0.76) | 35.39 (0.64) | 53.14 (0.82) | 60.15 (0.91) | Reference |

**Table S8. Ablation results on CG-TKG-8.**

| Variant | MRR, mean (SD) | Hits@1, mean (SD) | Hits@3, mean (SD) | Hits@10, mean (SD) | P value |
| --- | --- | --- | --- | --- | --- |
| w/o e | 50.53 (0.80) | 41.91 (0.74) | 55.95 (0.91) | 64.86 (0.98) | P<.001 |
| w/o r | 49.55 (0.84) | 40.04 (0.76) | 54.56 (0.93) | 62.35 (1.04) | P<.001 |
| w/o e&r | 34.49 (0.77) | 31.52 (0.70) | 36.56 (0.86) | 39.52 (0.96) | P<.001 |
| RL4TKGR (full) | 53.18 (0.79) | 45.48 (0.71) | 59.52 (0.88) | 67.26 (0.96) | Reference |

**Table S9. Ablation results on CG-TKG-12.**

| Variant | MRR, mean (SD) | Hits@1, mean (SD) | Hits@3, mean (SD) | Hits@10, mean (SD) | P value |
| --- | --- | --- | --- | --- | --- |
| w/o e | 54.30 (0.87) | 46.43 (0.79) | 63.54 (0.98) | 68.32 (1.08) | P<.001 |
| w/o r | 53.16 (0.90) | 44.72 (0.82) | 60.88 (1.01) | 66.39 (1.12) | P<.001 |
| w/o e&r | 42.95 (0.81) | 36.51 (0.73) | 44.76 (0.91) | 57.66 (1.00) | P<.001 |
| RL4TKGR (full) | 56.95 (0.84) | 49.75 (0.77) | 62.54 (0.93) | 71.79 (1.03) | Reference |

# 5. Fixed-Alpha Sensitivity Analysis

The revised main manuscript uses one merged figure to reduce redundancy. The original single-dataset plots are retained here as supplementary figures for traceability.

Figure S1. Fixed-alpha sensitivity analysis on CG-TKG-4.

Figure S2. Fixed-alpha sensitivity analysis on CG-TKG-4.

Figure S3. Fixed-alpha sensitivity analysis on CG-TKG-12.

# 6. Extended Case Analysis

Patient 49375 was selected as an illustrative case. The first visit occurred on June 22, 2013. Manifestations included occasional stomach pain after acute gastroenteritis, abdominal distension, poor belching, a foreign body sensation in the pharynx, phlegm in the pharynx, normal appetite, average sleep quality with many dreams, and normal urination and defecation. The tongue was dark red with a thin white coating, and the pulse was deep and thready. The diagnosis was chronic gastritis, and the prescribed medicines were Zhizhu Kuanzhong Capsules and Qingyan Tablets.

The second visit occurred on July 13, 2013. Manifestations included disappearance of stomach pain, occasional abdominal distension, a foreign body sensation in the pharynx, poor belching, phlegm in the pharynx, normal appetite, improved dreaminess, and normal urination and defecation. The diagnosis remained chronic gastritis, and the prescribed medicines were Zhizhu Kuanzhong Capsules and Qingyan Tablets.

The third visit occurred on August 3, 2013. Manifestations included loose stools in the previous week, occasional abdominal distension, poor belching, phlegm in the pharynx, normal appetite, average sleep quality with many dreams, and normal urination. RL4TKGR used the historical diagnostic information and third-visit symptom information to rank candidate medicines and disease diagnoses.

Table S10. Top 10 reasoning results for patient 49375 at the third visit.

| Rank | (Patient 49375, prescribe medicine, ', 3) | (Patient 49375, disease diagnosis, ', 3) |
| --- | --- | --- |
| 1 | Zhizhu Kuanzhong Capsules | Chronic gastritis |
| 2 | Guben Yichang Tablets | Chronic atrophic gastritis |
| 3 | Simo Decoction Oral Liquid | Chronic superficial gastritis |
| 4 | Bazhen Yimu Pills | Erosive gastritis |
| 5 | Qingyan Tablets | Helicobacter pylori-associated gastritis |
| 6 | Xuhanting Capsules | Spleen deficiency and dampness-heat syndrome |
| 7 | Wuweizi Granules | Mild dysplasia of chronic gastritis |
| 8 | Qizhi Weitong Granules | Chronic gastritis with intestinal metaplasia |
| 9 | Xuhan Weitong Granules | Chronic gastritis with hiatal hernia |
| 10 | Jinghua Weikang Capsules | Liver stagnation and spleen deficiency syndrome |

The fourth visit occurred on September 21, 2013. The patient showed marked clinical improvement, including alleviated abdominal distension, belching, reduced night sweats, general chills, disappearance of phlegm, normal appetite, average sleep, sometimes formed stools, and normal urination. The patient was diagnosed with chronic gastritis. This illustrative case supports the plausibility of the predicted medication ranking and indicates that RL4TKGR may help identify candidate therapies for clinical review.

# 7. CG-TKG Construction and Quality Control

This supplementary section provides additional detail on how the Chronic Gastritis Temporal Knowledge Graph (CG-TKG) was constructed from deidentified clinical records and how the graph construction process was checked for semantic consistency.

Source fields and entity extraction. Clinical entities were extracted from structured and semistructured fields, including symptoms, TCM diagnoses, Western medicine diagnoses, TCM syndrome diagnoses, prescriptions, laboratory tests, gastroscopy findings, and pathological findings. Entity normalization was performed according to the clinical field type so that synonymous or repeated expressions within the same record mapped to the corresponding entity category.

Relation construction. Relations were generated from the clinical role of each field in a visit-level record, including symptom presentation, disease diagnosis, TCM syndrome diagnosis, prescription, laboratory-test observation, gastroscopy finding, and pathological finding. These relations connect the anonymized patient or visit node to the corresponding medical entity.

Temporal encoding. The temporal index used in CG-TKG is the visit order rather than the calendar date. For a patient with multiple visits, visit 1, visit 2, and subsequent visits form the temporal sequence. This design avoids treating irregular follow-up intervals as evenly spaced calendar time while preserving the longitudinal order of diagnosis and treatment events.

Merging multisource observations within one visit. Symptoms, diagnoses, prescriptions, laboratory tests, gastroscopy findings, and pathological findings recorded during the same visit were merged into one visit-level temporal snapshot. All observed facts from the same visit share the same visit-order timestamp, and no artificial ordering was imposed among different data sources within that visit.

Handling missing or asynchronous observations. Missing clinical items were not imputed and were not treated as negative facts. Only observed clinical facts were retained in the graph. If a laboratory, gastroscopy, or pathological observation was recorded as part of a visit, it was assigned to the corresponding visit-order timestamp; otherwise, the absent item was simply not represented as a graph fact.

Expert review and quality control. TCM experts performed a full review of the entity and relation schema used to construct the CG-TKG. Generated graph facts were further checked through sampled triple-level and quadruple-level verification to assess whether the extracted entity, relation, and visit-order timestamp were semantically consistent with the source clinical record. Discrepancies identified during expert review were used to refine the mapping rules before final dataset construction. The expert verification was conducted as a consensus-based quality control process rather than as independent duplicate annotation; therefore, an inter-rater agreement statistic was not calculated.

# 8. Statistical Confidence Intervals and Computational Cost

This section supplements the main manuscript by reporting 95% confidence intervals for the primary MRR comparisons and by summarizing the computational environment, runtime, memory use, and convergence behavior of RL4TKGR.

Confidence intervals were calculated from five independent runs using mean +/- t0.975,4 x SD / sqrt(5), where t0.975,4 = 2.776. Because MRR is the primary ranking metric in the manuscript, the table below focuses on MRR confidence intervals.

Table S11. Primary MRR results with 95% confidence intervals.

| Analysis | Dataset | Model or variant | MRR, mean (SD) | 95% CI |
| --- | --- | --- | --- | --- |
| Comparative | CG-TKG-4 | DaeMon | 43.26 (0.95) | 42.08-44.44 |
| Comparative | CG-TKG-4 | RL4TKGR | 51.66 (0.76) | 50.72-52.60 |
| Comparative | CG-TKG-8 | DaeMon | 51.76 (1.12) | 50.37-53.15 |
| Comparative | CG-TKG-8 | RL4TKGR | 53.18 (0.79) | 52.20-54.16 |
| Comparative | CG-TKG-12 | DaeMon | 54.87 (1.17) | 53.42-56.32 |
| Comparative | CG-TKG-12 | RL4TKGR | 56.95 (0.84) | 55.91-57.99 |
| Ablation | CG-TKG-4 | w/o e | 44.05 (0.82) | 43.03-45.07 |
| Ablation | CG-TKG-4 | w/o r | 41.59 (0.88) | 40.50-42.68 |
| Ablation | CG-TKG-4 | w/o e&r | 33.54 (0.74) | 32.62-34.46 |
| Ablation | CG-TKG-4 | RL4TKGR (full) | 51.66 (0.76) | 50.72-52.60 |
| Ablation | CG-TKG-8 | w/o e | 50.53 (0.80) | 49.54-51.52 |
| Ablation | CG-TKG-8 | w/o r | 49.55 (0.84) | 48.51-50.59 |
| Ablation | CG-TKG-8 | w/o e&r | 34.49 (0.77) | 33.53-35.45 |
| Ablation | CG-TKG-8 | RL4TKGR (full) | 53.18 (0.79) | 52.20-54.16 |
| Ablation | CG-TKG-12 | w/o e | 54.30 (0.87) | 53.22-55.38 |
| Ablation | CG-TKG-12 | w/o r | 53.16 (0.90) | 52.04-54.28 |
| Ablation | CG-TKG-12 | w/o e&r | 42.95 (0.81) | 41.94-43.96 |
| Ablation | CG-TKG-12 | RL4TKGR (full) | 56.95 (0.84) | 55.91-57.99 |

Table S12. Computational cost and convergence summary for RL4TKGR.

| Dataset | Batches per epoch | Time per epoch | Total training time | Peak GPU memory | Peak system memory |
| --- | --- | --- | --- | --- | --- |
| CG-TKG-4 | ~316 | ~1.5-3 min | ~2-4 h | ~2-4 GB | ~4-8 GB |
| CG-TKG-8 | ~134 | ~40-80 s | ~1-2 h | ~2-3 GB | ~3-6 GB |
| CG-TKG-12 | ~72 | ~25-45 s | ~0.5-1 h | ~2-3 GB | ~3-5 GB |

The experiments were run on a workstation with one NVIDIA GeForce RTX 4090 GPU (24 GB), an Intel Core i7 CPU, and 16-32 GB system RAM. The model was trained for a maximum of 50-100 epochs with early stopping based on validation MRR and a patience of 5-10 epochs. In typical runs, convergence occurred within approximately 40-70 epochs. CG-TKG-12 converged earlier than CG-TKG-4 because it had fewer training quadruples and a more stable long-course visit structure.

# 9. Path-Level Interpretability of Patient A

This section supplements the main manuscript by providing the exported reasoning paths for the representative anonymized case, Patient A. Both queries are issued at the third visit. The event balance factor beta reports how strongly the final policy relies on historical evidence, whereas 1-beta represents the contribution assigned to non-historical or current-visit evidence.

Table S13. Historical evidence paths exported for Patient A.

| Query | Prediction (rank 1) | beta | Historical evidence path over CG-TKG | Interpretation |
| --- | --- | --- | --- | --- |
| Disease diagnosis, (Patient A, disease diagnosis, ', visit 3) | Chronic gastritis | 0.58 | Patient A -> [disease diagnosis, visit 1] -> Chronic gastritis -> [disease diagnosis, visit 2] -> Chronic gastritis -> [disease diagnosis, visit 3] -> ' | The two prior visits both resolve to chronic gastritis. The model follows this historical diagnostic chain and preserves diagnostic continuity at visit 3. A beta value above 0.5 indicates that the historical path dominates this decision. |
| Medication, (Patient A, prescribe medicine, ', visit 3) | Zhizhu Kuanzhong Capsules | 0.62 | Patient A -> [prescribe medicine, visit 1] -> Zhizhu Kuanzhong Capsules -> [prescribe medicine, visit 2] -> Zhizhu Kuanzhong Capsules -> [prescribe medicine, visit 3] -> ' | The same prescription appears in the first two visits and is reused by the model for the third-visit medication query. The higher beta value reflects stronger reliance on prescribing history. |

Table S14. Non-historical current-visit evidence paths for Patient A.

| Query | Non-historical evidence path at visit 3 | Contribution (1-beta) |
| --- | --- | --- |
| Disease diagnosis | Patient A -> [has symptom, visit 3] -> abdominal distension, poor belching, phlegm in the pharynx -> [symptom-of] -> chronic gastritis | 0.42 |
| Medication | Patient A -> [has symptom, visit 3] -> abdominal distension, loose stools -> [treated-by] -> Zhizhu Kuanzhong Capsules | 0.38 |

Table S15. Patient-level counterfactual check after masking the historical path.

| Query | Full model, rank of true entity | Historical path masked (beta=0) |
| --- | --- | --- |
| Disease diagnosis | 1 | 4; erosive gastritis ranked above the true entity |
| Medication | 1 | 5; Qingyan Tablets ranked above the true entity |

These patient-level checks show that the rank-1 predictions are not supported only by top-ranking output. They can be traced to exported historical paths, current-visit symptom paths, and counterfactual rank changes when historical evidence is masked.

# 10. TCM Terminology Glossary

This glossary is provided to help international readers interpret the TCM-specific terms used in the manuscript, CG-TKG construction, and case analysis. The definitions are intended for reader orientation and do not replace formal clinical definitions.

| Term | Explanation |
| --- | --- |
| Traditional Chinese Medicine (TCM) | A medical system that uses syndrome differentiation and individualized diagnosis and treatment based on symptoms, signs, and other clinical observations. |
| CG-TKG | The chronic gastritis temporal knowledge graph constructed in this study from deidentified longitudinal diagnosis and treatment records. |
| TCM diagnosis | A diagnosis or clinical judgement recorded under the TCM diagnostic framework. |
| TCM syndrome diagnosis | A pattern-based diagnostic category in TCM, such as spleen-stomach weakness or liver-stomach disharmony, rather than a Western disease label. |
| Syndrome differentiation | The TCM process of identifying a patient-level pattern from clinical manifestations, including symptoms, tongue appearance, pulse condition, and other observations. |
| Four diagnostic methods | The traditional TCM diagnostic approaches of inspection, auscultation-olfaction, inquiry, and palpation. |
| Chief complaint | The patient's main symptom or reason for consultation at a visit. |
| Present illness history | Narrative information describing symptom onset, duration, progression, previous treatment, and current clinical status. |
| Tongue coating | The observed layer on the tongue surface, which is recorded as one TCM diagnostic feature. |
| Pulse condition | A TCM examination finding based on palpation of pulse characteristics. |
| Symptom entity | A standardized graph entity representing a recorded symptom or clinical manifestation, such as gastric distension, stomach pain, acid regurgitation, poor appetite, or heartburn. |
| Treatment entity | A standardized graph entity representing a recorded treatment, including prescriptions, Chinese patent medicines, or other medication names retained in the clinical records. |

# 11. RL4TKGR Training and Inference Pseudocode

Algorithm S1. RL4TKGR training and inference workflow.

This pseudocode summarizes the procedural workflow of RL4TKGR, linking the dual-channel reward function, dual-path policy network, event balance factor beta, action selection, reward aggregation, and REINFORCE update.

| Step | Procedure |
| --- | --- |
| 1 | Input: training quadruples F_train, query q=(s,p,?,t), maximum path length L, and candidate action sets A_n. |
| 2 | Initialize entity, relation, and timestamp embeddings; initialize policy network parameters theta and dynamic weight allocator parameters phi that produce beta. |
| 3 | For each training query, initialize the state s_0=(e_q,t_q,e_q,t_q,r_q) and an empty reasoning path. |
| 4 | At step n, construct the available action set A_n and encode the current diagnosis representation e_nt using the entity and visit-number embeddings. |
| 5 | Encode the historical path with the LSTM branch to obtain h_n^his and encode the non-historical information with the MLP branch to obtain h_n^nhis. |
| 6 | Compute the event balance factor beta from the query representation and combine the two action distributions as P=beta P_his+(1-beta)P_nhis. |
| 7 | Sample or select action a_n according to P, update the state to s_{n+1}, and append the selected action to the reasoning path. |
| 8 | After L steps, compute R_his and R_nhis, and aggregate them as R=alpha R_his+(1-alpha)R_nhis with alpha provided by beta. |
| 9 | Update theta and phi using the REINFORCE policy-gradient objective based on the aggregated reward R. |
| 10 | Output: during inference, rank candidate diagnosis or treatment entities according to the learned policy scores and return the top-ranked predictions. |

| **Algorithm 1.** Training and inference workflow of RL4TKGR | |
| --- | --- |
| **Input:** training quadruple set *F*_train_; validation set *F*_valid_; test query set *Q*_test_; query *q* = (*e*_q_, *r*_q_, ?, *t*_q_); maximum reasoning path length *L*; number of epochs *N*_e_; batch size *B*; learning rate *η* | |
| **Output:** optimized parameters (*θ**, *φ**); for every *q* ∈ *Q*_test_, the top-*k* ranked candidate diagnosis or therapeutic drug entities | |
| **Phase 1: Training — joint optimization of *θ* and *φ*** | |
| 1: | Initialize entity embeddings *e*, relation embeddings *r*, and visit number (timestamp) embeddings *t* of dimensions *d*_e_, *d*_r_, and *d*_t_ by uniform sampling |
| 2: | Initialize policy network parameters *θ* and dynamic weight allocator parameters *φ* = {*W*, *b*}; *patience* ← 0 |
| 3: | **for** *epoch* = 1 **to** *N*_e_ **do** |
| 4: | **for each** mini-batch *B*′ ⊆ *F*_train_ of size *B* **do** |
| 5: | **for each** query *q* = (*e*_q_, *r*_q_, ?, *t*_q_) ∈ *B*′ with ground-truth object *e*_fact_ **do** |
| 6: | *s*_0_ ← (*e*_q_, *t*_q_, *e*_q_, *t*_q_, *r*_q_); *τ* ← ∅; *a*_0_ ← (*r*_q_, *e*_q_, *t*_q_); *h*_0_^his^ ← LSTM(0, *E*(*a*_0_)) *▷ initial state, empty reasoning path, start action* |
| 7: | *H*_q_ ← entities observed in the *n* visits preceding *t*_q_; compute the historical occurrence frequencies *p*_his_(·) over *H*_q_ |
| 8: | *v*_q_ ← [*s*_q_; *p*_q_; *t*_q_]; *β* ← sigmoid(*W* · *v*_q_ + *b*) *▷ query-level event balance factor, Eq. 9* |
| 9: | **for** *n* = 0 **to** *L* − 1 **do** |
| 10: | *A*_n_ ← {(*r*′, *e*′, *t*′) \| (*e*_n_, *r*′, *e*′, *t*′) ∈ *F*_t′_, *t*′ < *t*_q_} *▷ available action set at step n* |
| 11: | Δ*t* ← *t*_q_ − *t*_n_; *e*_n_^t^ ← [*e*_n_; *σ*(*w*Δ*t* + *b*)] *▷ diagnosis embedding module, Eq. 4* |
| 12: | **if** *n* > 0 **then** *h*_n_^his^ ← LSTM(*h*_n−1_^his^, *E*(*a*_n−1_)) **end if** *▷ LSTM historical branch, Eq. 6* |
| 13: | **for each** candidate *a* ∈ *A*_n_ **do** *h*^nhis^(*a*) ← *W*_2_ · ReLU(*W*_1_ · [*s*; *p*; *E*(*a*)] + *b*_1_) + *b*_2_ **end for** *▷ MLP non-historical branch, Eq. 7* |
| 14: | *P*_his_ ← softmax_a ∈ An_ *f*_his_(*h*_n_^his^, *e*_n_^t^, *E*(*a*)); *P*_nhis_ ← softmax_a ∈ An_ *f*_nhis_(*h*^nhis^(*a*)) *▷ action scoring module* |
| 15: | *P* ← *β* · *P*_his_ + (1 − *β*) · *P*_nhis_ *▷ dual-path action distribution, Eq. 8* |
| 16: | *a*_n_ ∼ *P* *▷ stochastic action selection during training* |
| 17: | *s*_n+1_ ← *δ*(*s*_n_, *a*_n_) = (*e*_n+1_, *t*_n+1_, *e*_q_, *t*_q_, *r*_q_); append *a*_n_ to *τ* *▷ state transition* |
| 18: | **end for** |
| 19: | *e*_L_ ← terminal entity of the state *s*_L_ reached along the path *τ* |
| 20: | *R*_his_ ← *I*{*e*_L_ = *e*_fact_ ∧ *e*_L_ ∈ *H*_q_} · (1 + *p*_his_(*e*_L_)) *▷ historical reward, Eq. 1* |
| 21: | *R*_nhis_ ← *I*{*e*_L_ = *e*_fact_} *▷ non-historical reward, Eq. 2* |
| 22: | *α* ← *β*, treated as a constant with no gradient through the reward; *R* ← *α* · *R*_his_ + (1 − *α*) · *R*_nhis_ *▷ dual-channel aggregated reward, Eq. 3* |
| 23: | **end for** |
| 24: | ∇_θ,φ_*J* ← *E*_πθ,φ_[∇_θ,φ_ log *π*_θ,φ_(*a*_n_ \| *s*_n_) · *R*] *▷ REINFORCE policy gradient, Eqs. 10 and 11* |
| 25: | (*θ*, *φ*) ← Adam((*θ*, *φ*), ∇_θ,φ_*J*, *η*) *▷ joint update of policy network and weight allocator* |
| 26: | **end for** |
| 27: | Evaluate MRR on *F*_valid_; **if** MRR improves **then** (*θ**, *φ**) ← (*θ*, *φ*); *patience* ← 0 **else** *patience* ← *patience* + 1 **end if** |
| 28: | **if** *patience* > *patience*_max_ **then break end if** *▷ early stopping* |
| 29: | **end for** |
| **Phase 2: Inference — time-aware filtered link prediction** | |
| 30: | **for each** test query *q* = (*e*_q_, *r*_q_, ?, *t*_q_) ∈ *Q*_test_ **do** |
| 31: | Execute lines 6–18 under the learned policy *π*_θ*,φ*_, replacing the sampling in line 16 by expansion of the highest-probability actions |
| 32: | *score*(*o*) ← aggregated probability of the trajectories under *π*_θ*,φ*_ that terminate at candidate entity *o* |
| 33: | Discard every *o*′ ≠ *e*_fact_ such that (*e*_q_, *r*_q_, *o*′, *t*_q_) is a known quadruple *▷ time-aware filtered setting* |
| 34: | Rank the remaining candidates by *score*(*o*) in descending order |
| 35: | **end for** |
| 36: | **return** the top-*k* ranked chronic gastritis diagnosis or therapeutic drug entities for every *q* ∈ *Q*_test_ |
